# Supplementary material for: Stress granule phase separation in stress-responsive cytosolic extract-in-oil droplets
Source: Nat Commun. 2026 Jun 5;17:5011. doi: 10.1038/s41467-026-73936-x (PMC13241516; doi:10.1038/s41467-026-73936-x)

Supplementary Information to

Stress granule phase separation in stress-responsive cytosolic extract-in-oil droplets.

Aline Lieber[1], Oskar Staufer[2,3], Zhaozhi Sun[1], Ulrike Engel[4], Charlotte Flory[5], Nathan Mikhaylenko[3], Kevin Jahnke[2], Katja Kopp[1], Philipp Klein[1], Sarah Hofmann[6], Oliver T. Fackler[7], Pavel Ivanov[6], Ilia Platzman[2], Pietro Scaturro[5], Joachim P. Spatz[2], and Alessia Ruggieri[1*].

Affiliations

[1] Department of Infectious Diseases, Molecular Virology, Center for Integrative Infectious Diseases Research, Heidelberg University, Medical Faculty Heidelberg, Heidelberg, Germany.

[2] Department for Cellular Biophysics, Max Planck Institute for Medical Research, Heidelberg, Germany; Institute for Molecular Systems Engineering (IMSE), Heidelberg University, Heidelberg, Germany; Max Planck-Bristol Centre for Minimal Biology, University of Bristol, Bristol, UK.

[3] INM - Leibniz Institute for New Materials, Saarbrücken, Germany.

[4] Nikon Imaging Center at Heidelberg University and Centre for Organismal Studies (COS), Heidelberg University, Heidelberg, Germany.

[5] Research group Systems Arbovirology, Leibniz Institute of Virology (LIV), Hamburg, Germany.

[6] Brigham and Women's Hospital, Harvard Medical School, Harvard Initiative for RNA Medicine, Boston, MA, USA.

[7] Department of Infectious Diseases, Integrative Virology, Center for Integrative Infectious Diseases Research, Heidelberg University, Medical Faculty Heidelberg, Germany.

Correspondence to

* alessia.ruggieri@med.uni-heidelberg.de

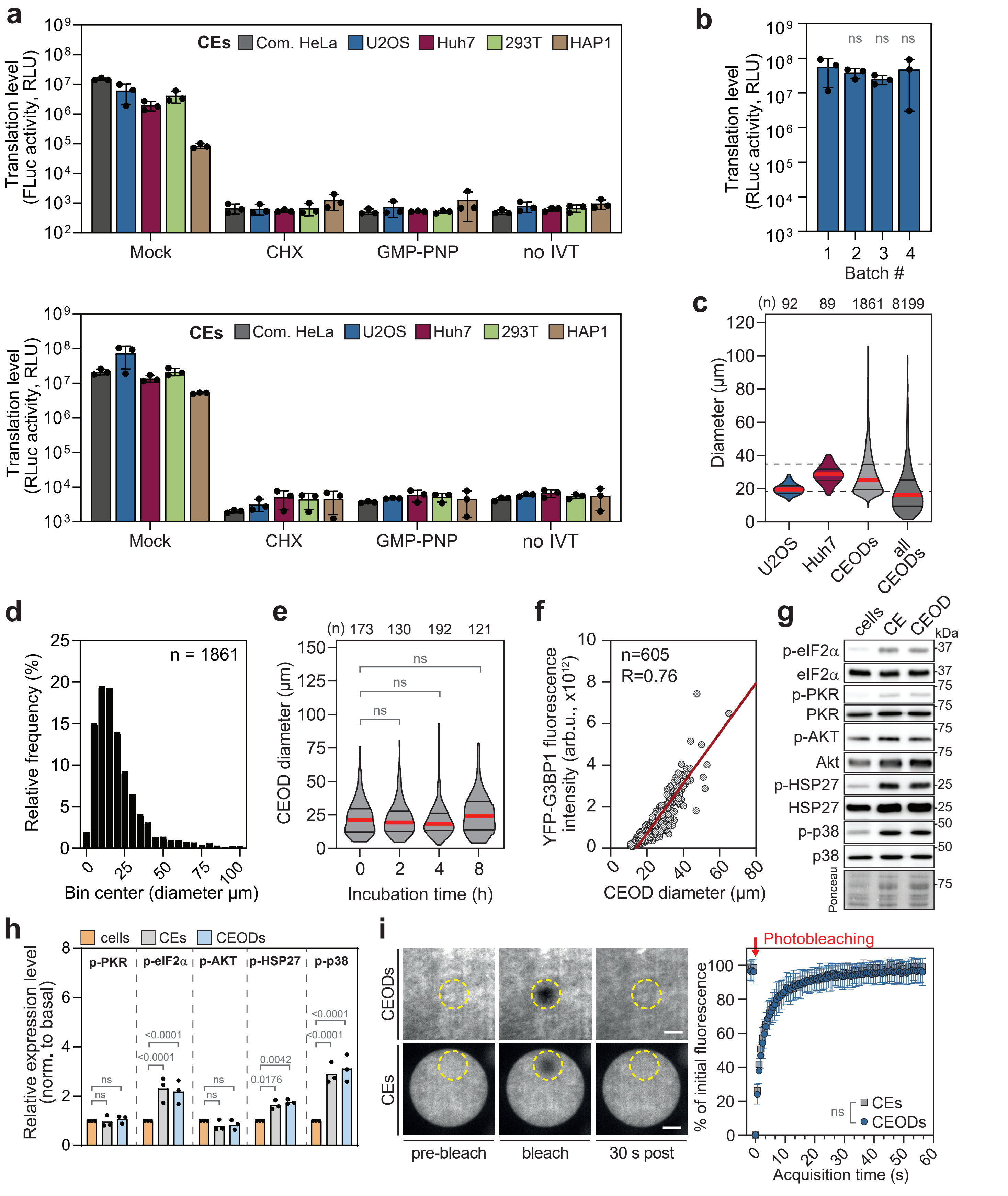

Supplementary Fig. 1. Characterisation of translation-competent CEODs. (**a**) Translation levels in CEs generated from U2OS, Huh7, 293T and HAP1 cells were compared to those of commercial HeLa extracts using reporter transcripts expressing firefly (FLuc, upper panel) and *Renilla* (RLuc, lower panel) luciferase transcripts. CEs treated with CHX and GMP-PNP served as negative control. Values (mean ± SD) correspond to the luciferase activity and are expressed as relative light units (RLU). (**b**) Translation levels of independent U2OS CE batches. Statistical significance (one-way ANOVA, Dunnett's mutipe comparisons test) compared to CE batch #1 is indicated. ns, non-significant ($p \geq 0.05$). (**c**) The diameter of the produced CEODs (CEOD all) was compared to that of U2OS and Huh7 cells in suspension. CEODs with a diameter above 15 µm were used for further analyses (CEODs). (**d**) CEOD diameter size distribution. Shown is the relative frequency and the number (n) of analysed CEODs produced by mechanical agitation. (**e**) Impact of time on CEOD diameter upon incubation at 37°C. The number of analysed CEODs (n) and statistical significance (Kruskal-Wallis test, Dunn's multiple comparison test) compared to time 0 h are indicated at the top. (**f**) Correlation between YFP-G3BP1 signal intensity and CEOD diameter. The number of analysed CEODs (n) and Pearson's R correlation coefficient (R) are indicated. (g and h) Expression level analysis of basal and phosphorylated (p-) forms of PKR, eIF2α, HSP27 and p38 in U2OS cells, CEs and CEODs (n=3 independent biological replicates). (**g**) Representative Western blot. (**h**) Corresponding quantifications (2-way ANOVA, Dunnett's multiple comparisons test). Protein levels were normalised to the loading control (Ponceau stain). arb.u., arbitrary units. (**i**) YFP-G3BP1 mobility in CEs and CEODs was assessed by FRAP measurements. A region of interest (yellow dotted circle) was photobleached for 5 s. The recovery of the YFP signal was measured for 60 s. Shown are representative images before, during and after photobleaching. Scale bar, 20 µm (left panel). Relative levels (% to YFP-G3BP1 fluorescence signal before photobleaching) is shown in the right panel (mean ± SD). 14 condensates and CEs were analysed per condition. Statistical significance (unpaired, two-sided Student's t-test) relative to CEs is indicated. ns, non-significant ($p \geq 0.05$).

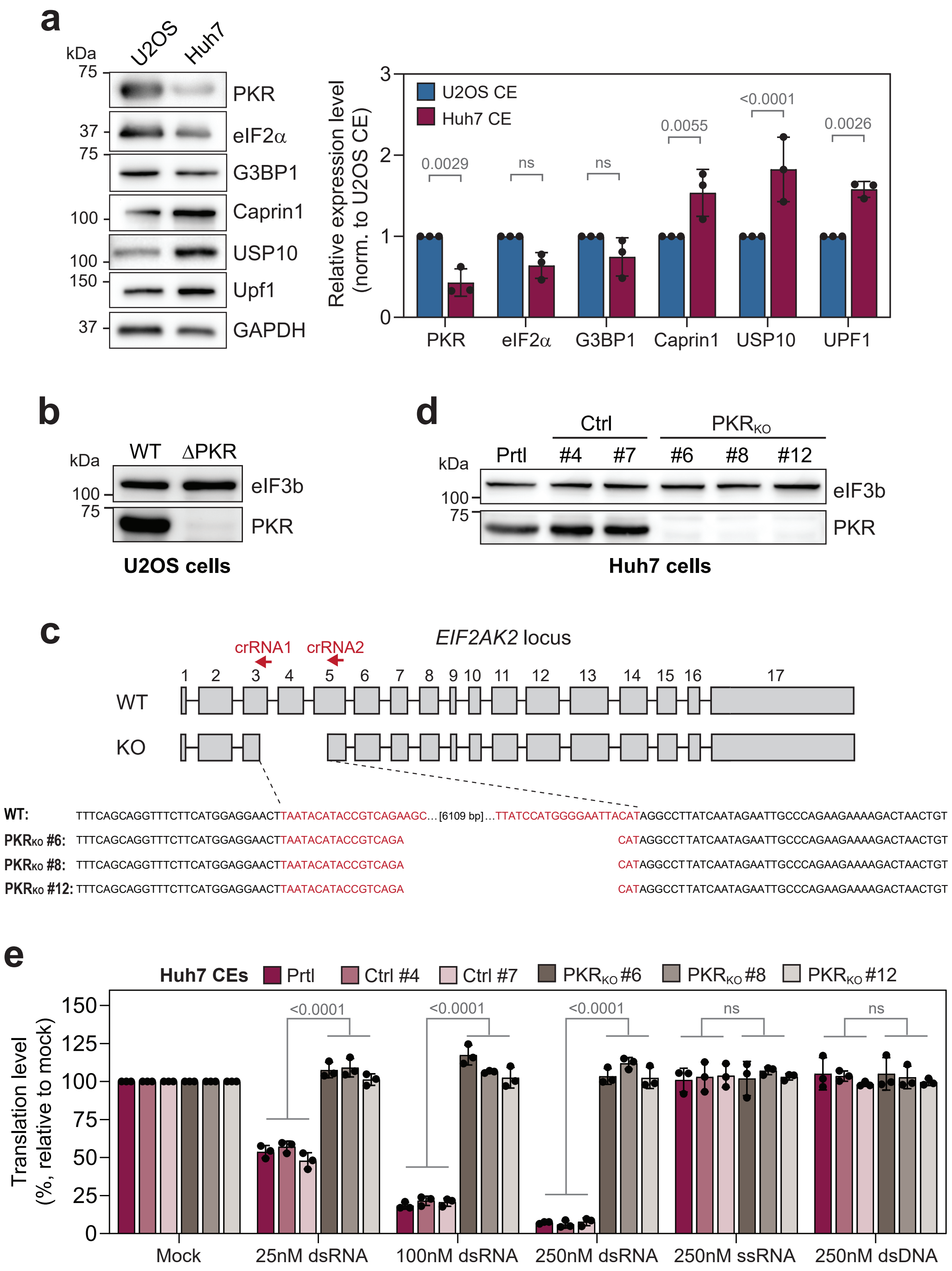

Supplementary Fig. 2. **PKR drives translation inhibition in response to dsRNA in CEs.** (**a**) Expression levels of ISR and SG components in U2OS and Huh7 cells (n = 3). Shown is a representative Western blot (left panel) and corresponding quantifications (right panel). Protein levels were normalised to the loading control GAPDH (arb.u., arbitrary units). Statistical significance (2-way ANOVA, Sidak's multiple comparisons test) compared to U2OS CE is indicated. (**b**) Analysis of PKR expression levels in U2OS and U2OS ΔPKR cells. (**c** to **e**) Generation of Huh7 PKR_{KO} cell clones. (c) Schematic of the human *EIF2AK2* locus (WT) encoding PKR. A sequence spanning exon 3 to 5 was deleted using two CRISPR guide RNAs (crRNA1 and crRNA2) (PKR_{KO}). Three homozygous clones were selected based on the deletion of the genomic DNA sequence between the guide RNAs and confirmed by sequencing. Dashes indicate nucleotide deletions. (d) PKR expression levels in Huh7 and Huh7 PKR_{KO} cells. (e) Relative translation levels in CEs produced from Huh7 parental CE, Huh7 non-targeting control cell clones (Ctrl) and Huh7 PKR_{KO} cell clones (n=3 independent biological replicates). CEs were treated with increasing concentrations of dsRNA. Values (mean ± SD) are represented as percentage relative to mock. CEs treated with ssRNA and dsDNA served as control. Statistical significance (2-way ANOVA, Tukey's multiple comparisons test) compared to Ctrl clones is indicated. ns, non-significant ($p \geq 0.05$).

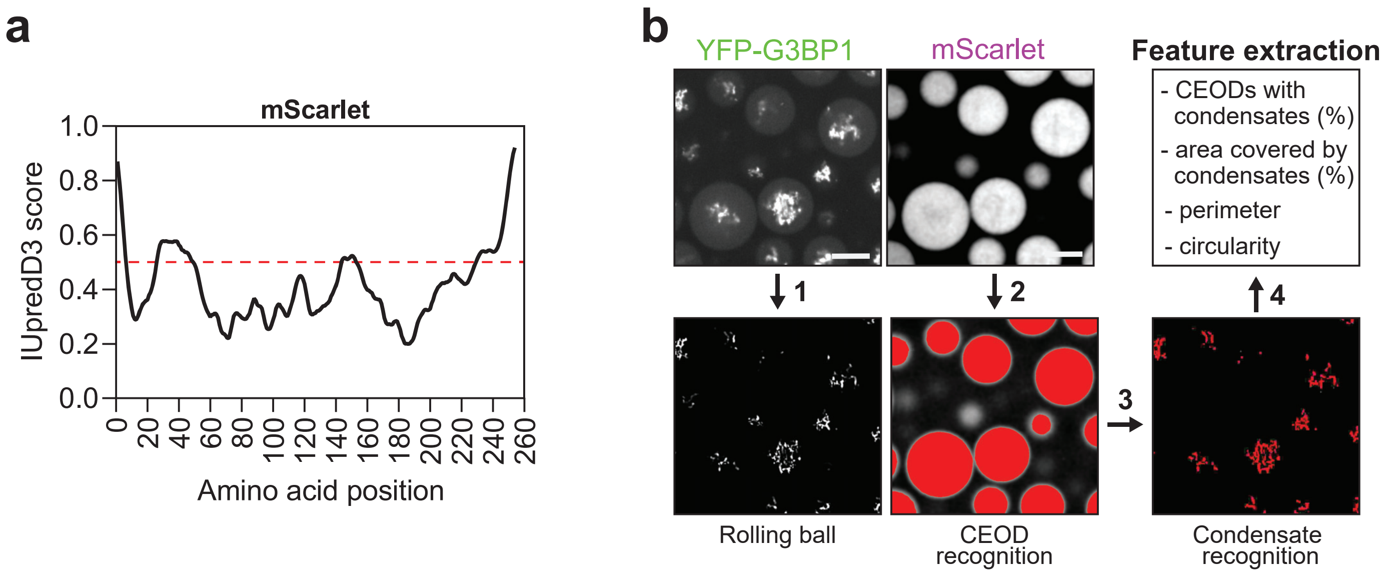

Supplementary Fig. 3. Image analysis of CEODs and condensates. (**a**) The fluorescent mScarlet protein was used for the segmentation of CEODs. Shown is the prediction of intrinsically unstructured regions in mScarlet amino acid sequence as analysed by the online tool IUPred3 (https://iupred3.elte.hu/). Values above the red dotted line indicate disordered regions. (**b**) Image analysis pipeline designed for the detection and analysis of CEOD and condensate features. Images were taken at the CEOD midsection and analysed using 2D-analysis.

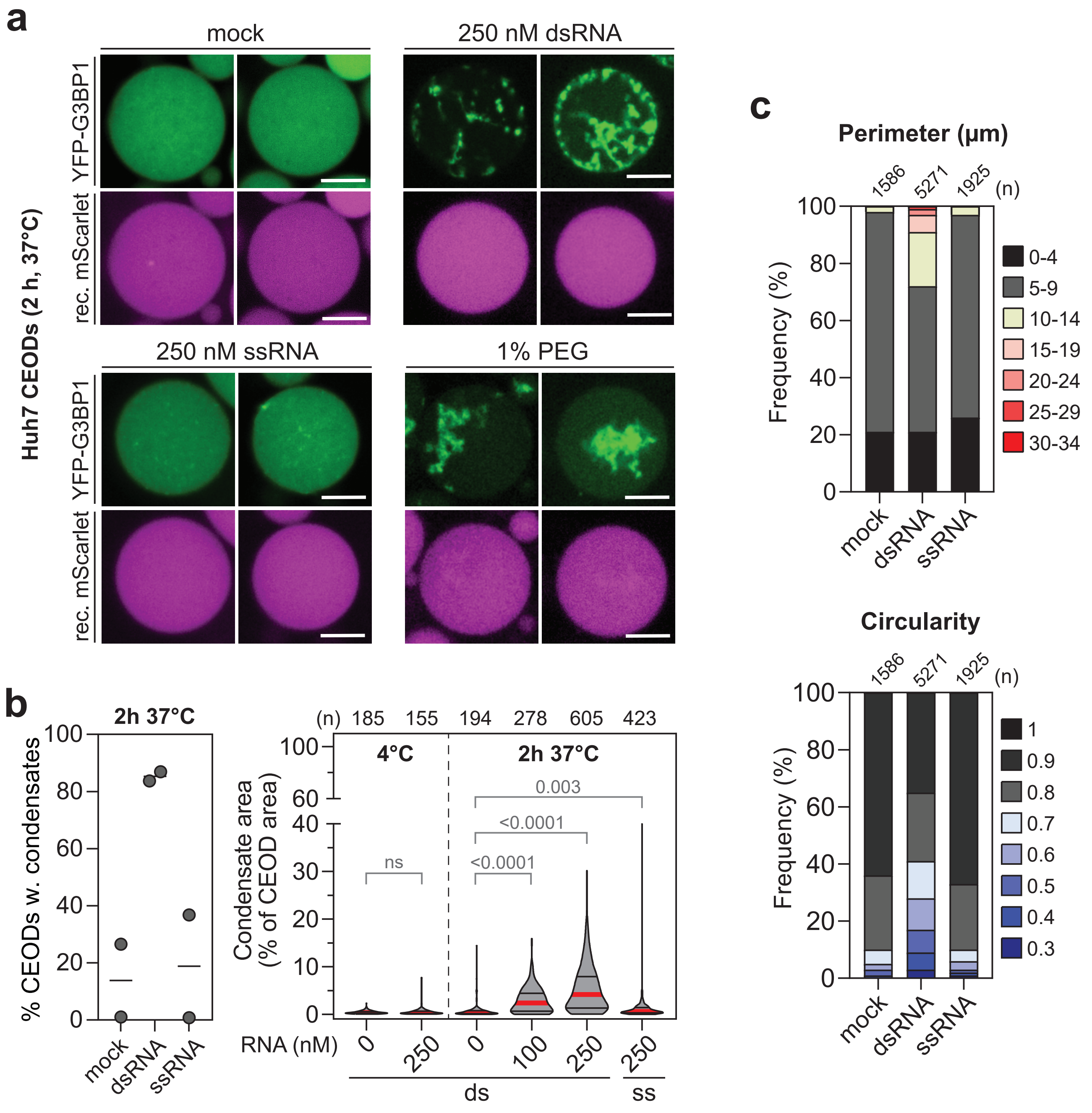

Supplementary Fig. 4. dsRNA-induced condensation of YFP-G3BP1 in Huh7 CEODs. (**a**) Representative images of Huh7 YFP-G3BP1 CEODs treated with 250 nM dsRNA, 250 nM ssRNA, 1% PEG or left untreated (mock). Scale bar, 20 µm. (**b** and **c**) Corresponding analyses of YFP-G3BP1 condensate area and condensate features. (b) Percentage of CEODs with YFP-G3BP1 condensates (left panel, 2 biological replicates) and median YFP-G3BP1 condensate area (right panel). The number of analysed CEODs (n) from 2 biological replicates and statistical significance (Kruskal-Wallis test, Dunn's multiple comparison test) compared to 0 nM dsRNA (ds) are indicated. (c) Bar graphs show the frequency of condensates with specified perimeter (top panel) and circularity (bottom panel). The number of analysed CEODs (n) from 2 biological replicates is indicated on the top. ns, non-significant ($p \geq 0.05$).

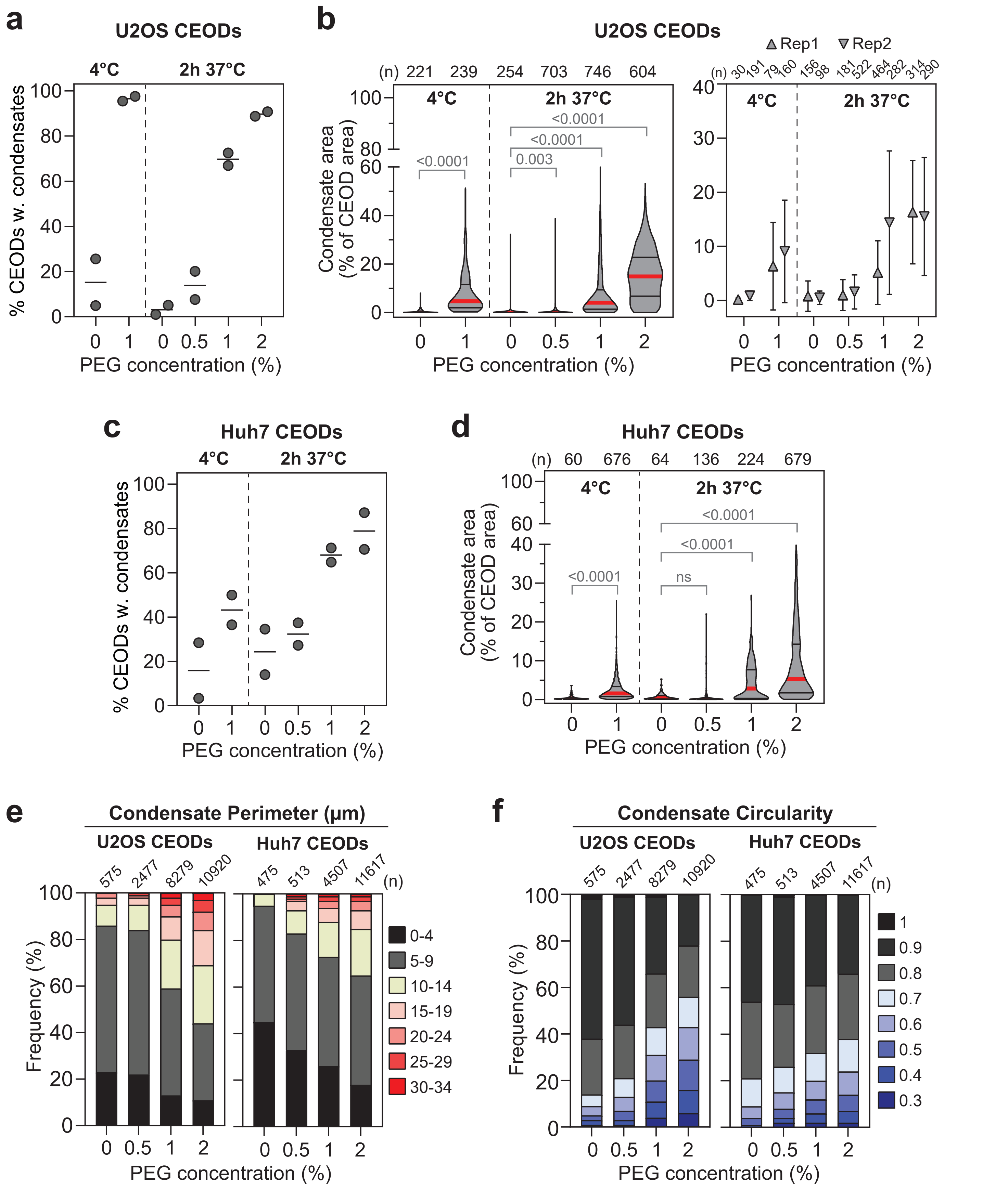

Supplementary Fig. 5. PEG-induced condensation of YFP-G3BP1 in CEODs. U2OS and Huh7 CEODs were treated with increasing concentrations of PEG. (**a**) Percentage of U2OS CEODs with YFP-G3BP1 condensates at 4°C and 37°C (2 biological replicates). (**b**) Analysis of U2OS YFP-G3BP1 condensates formed at 4°C and 37°C. Left panel: median condensate area, i.e. percentage of the CEOD area covered by condensates. The number of analysed CEODs (n) for 2 biological replicates and statistical significance (Kruskal-Wallis test, Dunn's multiple comparison test) compared to 0% PEG (untreated) are indicated. Right panel: mean ±SD condensate area for each biological replicate (Rep). The number of analysed CEODs (n) for each biological replicate is indicated on the top. (**c**) Percentage of Huh7 CEODs with YFP-G3BP1 condensates at 4°C and 37°C. (**d**) Median Huh7 YFP-G3BP1 condensate area. The number of analysed CEODs (n) from 2 biological replicates and statistical significance (Kruskal-Wallis test, Dunn's multiple comparison test) compared to 0% PEG are indicated. (**e** and **f**) Features of PEG-induced YFP-G3BP1 condensates. Bar graphs show the frequency of condensates with specified perimeter (e) and circularity (f). The number of analysed CEODs (n) from 2 biological replicates is indicated on the top.

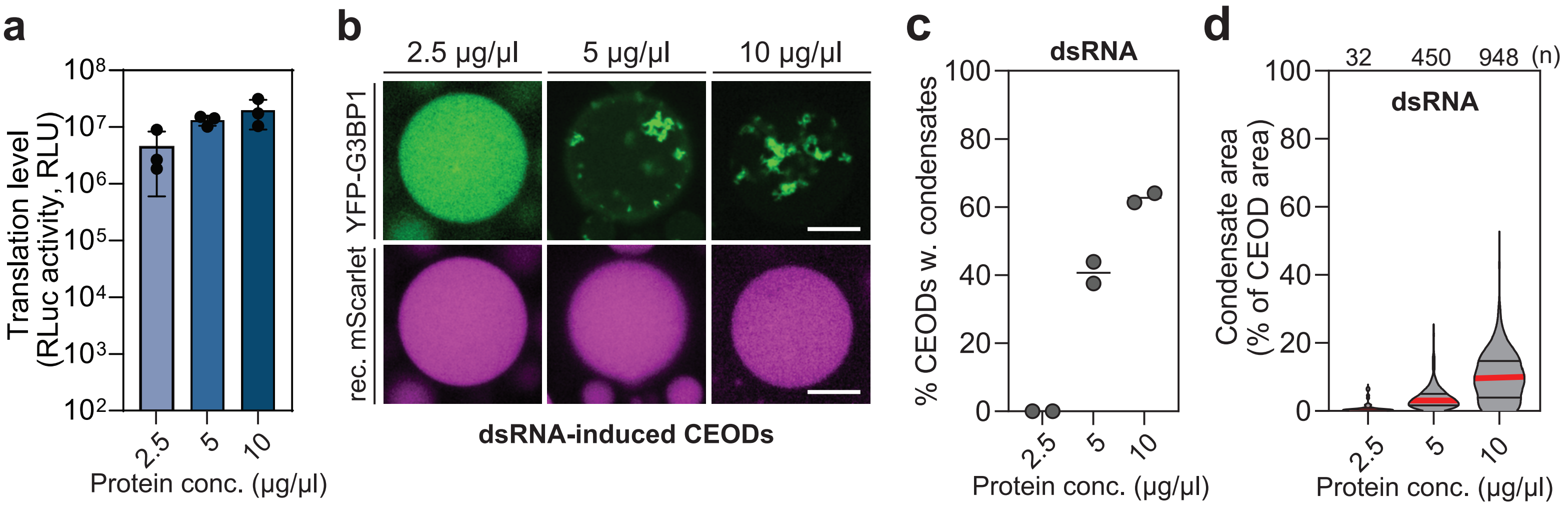

Supplementary Fig. 6. Influence of protein concentration on YFP-G3BP1 LLPS in CEODs. U2OS YFP-G3BP1 CEs were produced with a final protein concentration of 2.5, 5 or 10 µg/µl (n=3 independent biological replicates). (**a**) Translation levels (mean ± SD) of RLuc transcript in U2OS CEs with increasing protein concentrations. (**b** to **d**) Analysis of condensate formation. (b) Representative images of U2OS YFP-G3BP1 CEODs prepared with CEs with indicated protein concentrations and treated with 250 nM dsRNA for 2 h at 37°C. Scale bar, 20 µm. (c) Percentage of CEODs with YFP-G3BP1 condensates (2 biological replicates). (d) Median YFP-G3BP1 condensate area. The number of analysed CEODs (n) from 2 biological replicates is indicated.

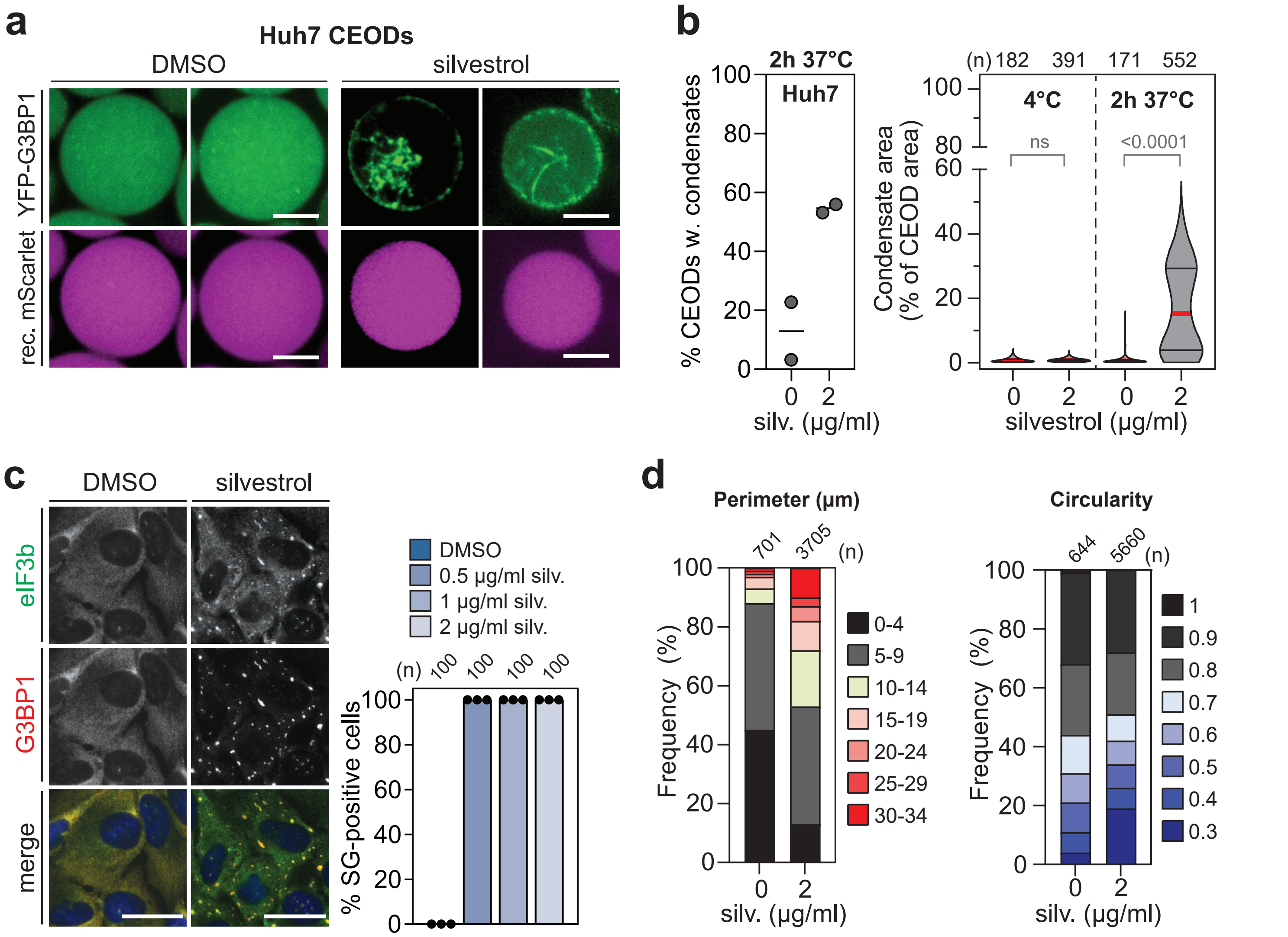

Supplementary Fig. 7. Inhibition of eIF4A by silvestrol induces YFP-G3BP1 condensation. (**a**) Representative images of Huh7 YFP-G3BP1 CEODs treated with vehicle control (DMSO) or 2 µg/ml silvestrol for 2 h at 37 °C. Scale bar, 20 µm. (**b**) Percentage of CEODs with YFP-G3BP1 condensates (left panel, 2 biological replicates) and median YFP-G3BP1 condensate area (right panel). The number of analysed CEODs (n) from 2 biological replicates and statistical significance (Kruskal-Wallis test, Dunn's multiple comparison test) compared to 0 µg/ml silvestrol are indicated. (**c**) Induction of SGs in U2OS cells treated with increasing concentrations of silvestrol (n=3 independent biological replicates). Shown are representative images (left panel) and quantifications (right panel). G3BP1 and eIF3B were stained as bona fide SG markers. Scale bar 50 µm. The number of analysed cells (n) is indicated on the top. (**d**) Features of silvestrol-induced YFP-G3BP1 condensates. Bar graphs show the frequency of condensates with specified perimeter (left panel) and circularity (right panel). The number of analysed CEODs (n) from 2 biological replicates is indicated on the top. ns, non-significant ($p \geq 0.05$).

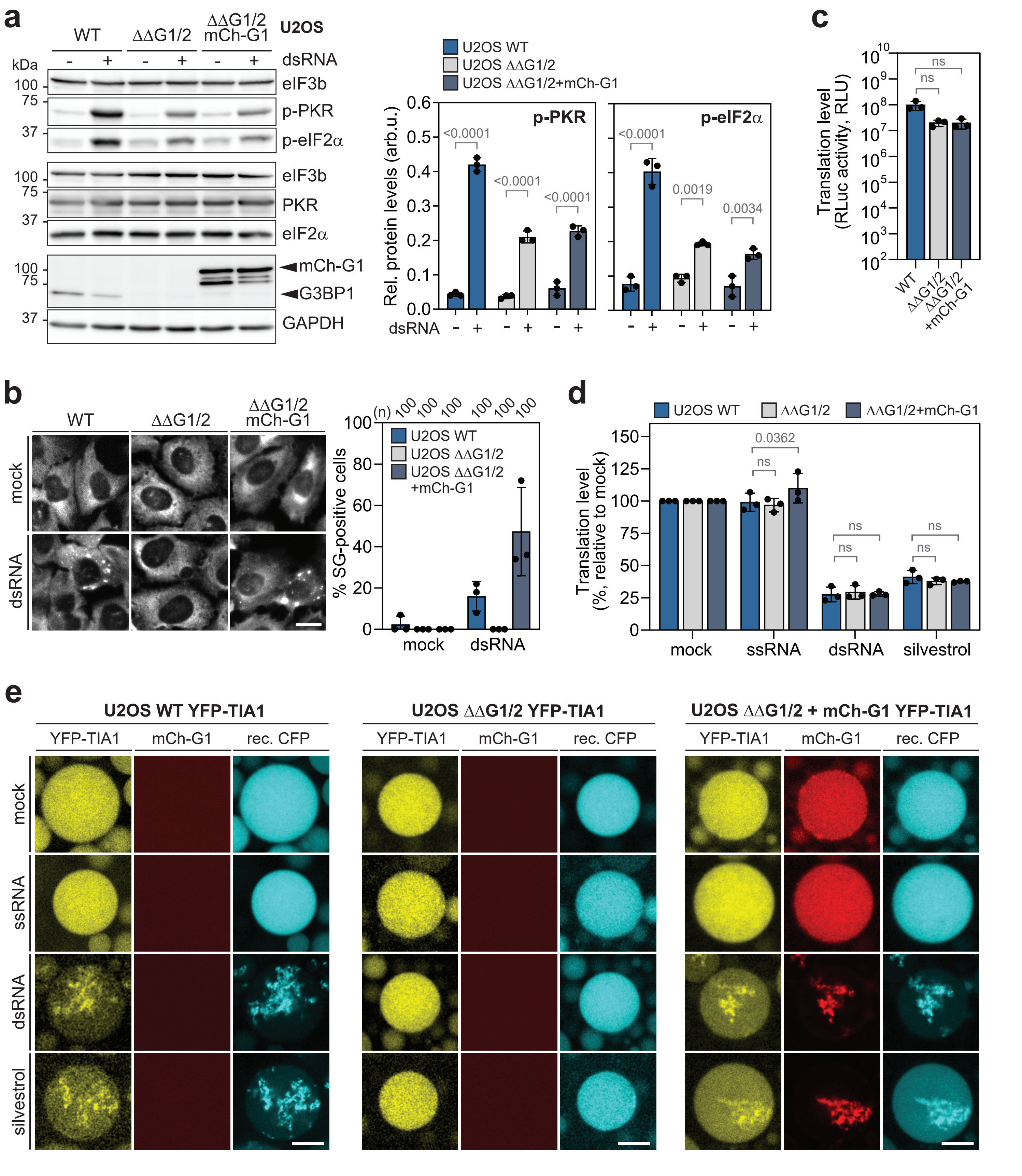

Supplementary Fig. 8. G3BP1 is essential for the formation of condensates in CEODs. The SG marker YFP-TIA1 was stably expressed in U2OS WT, U2OS ΔΔG1/2 and U2OS ΔΔG1/2 mCh-G1 cells. (**a**) Activation of the ISR by dsRNA was analysed in the different stable cells (n=3 independent biological replicates). Shown is a representative Western blot (left panel) and corresponding quantifications of p-PKR and p-eIF2α. Protein levels were normalised to the loading control eIF3b (right panel). arb.u., arbitrary units. Statistical significance (2-way ANOVA, Tukey's multiple comparisons test) compared to – dsRNA is indicated. (**b**) SG formation in all USOS cell lines was assessed by staining eIF3b in unstressed (mock) and dsRNA-treated cells. Left panel: representative images (scale bar 20 µm). Right panel: Corresponding quantifications (n=3 independent biological replicates). The number of cells (n) analysed is indicated on the top. (**c**) Translation levels of RLuc transcript in CEs generated from U2OS WT, U2OS ΔΔ G1/2 and U2OS ΔΔG1/2 mCh-G1 cells (n=3 independent biological replicates). Values (mean ± SD) correspond to the Rluc activity and are expressed as relative light units (RLU). Statistical analysis (1-way ANOVA, Tukey's multiple comparisons test) compared to U2OS WT is indicated. (**d**) Relative translation levels in CEs generated from U2OS WT, U2OS ΔΔG1/2 and U2OS ΔΔG1/2 mCh-G1 cells, treated with 250 nM ssRNA, 250 nM dsRNA or 2 µg/ml silvestrol. Values (mean ± SD) are represented as percentage relative to non-treated CEs (mock). Statistical analysis (2-way ANOVA, Tukey's multiple comparisons test) compared to U2OS WT is indicated. (**e**) CEODs were generated from all cell lines and treated for 2 h with ssRNA, dsRNA or silvestrol. A recombinant cyan fluorescent protein (rec. CFP) was spiked in the CEs before encapsulation for visualisation of the CEODs. Shown are representative images (scale bar, 20 µm). The appearance of condensation was visualised by analysing YFP-TIA1 fluorescence signal. Note that rec. CFP also accumulated in YFP-TIA1 condensates due to its high level of IDRs. ns, non-significant ($p \geq 0.05$).

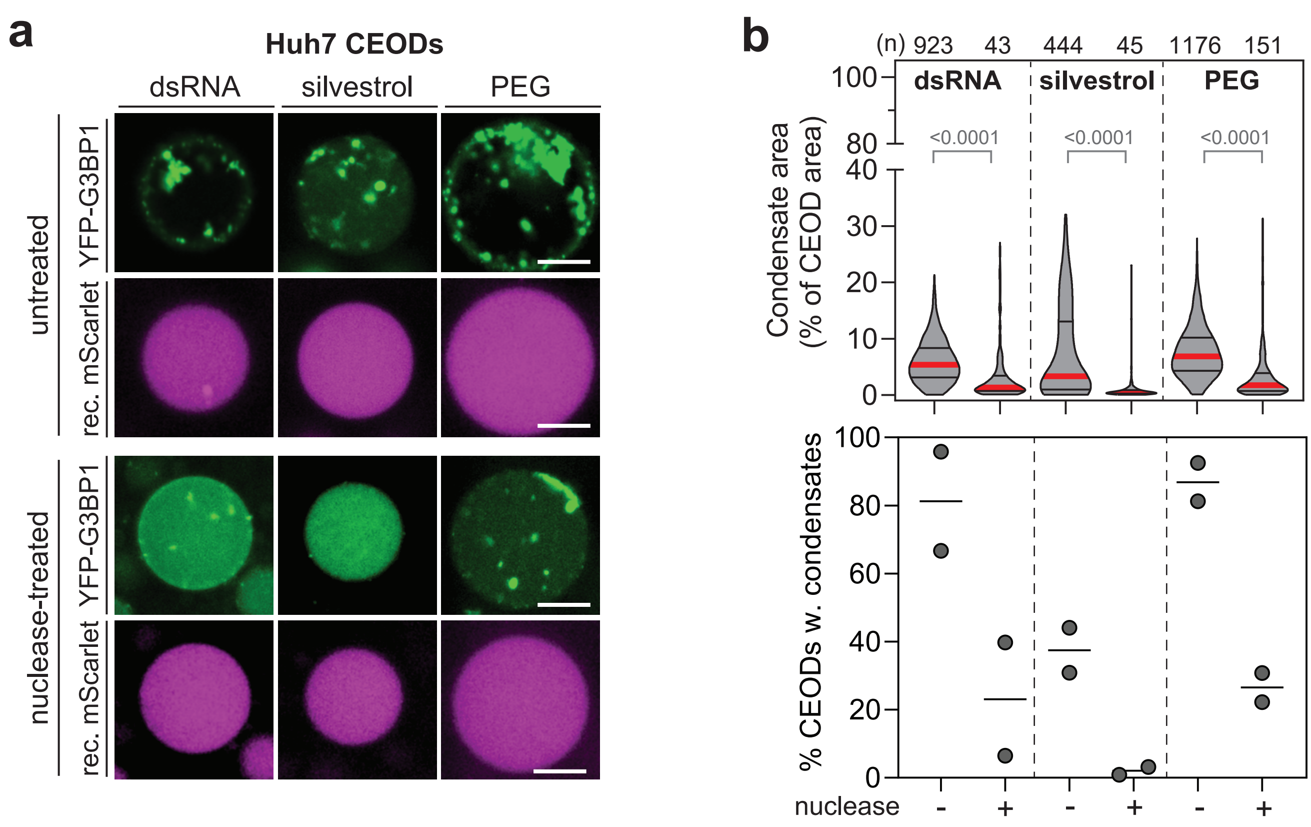

Supplementary Fig. 9. Endogenous RNAs are required for YFP-G3BP1 condensation in CEODs. Huh7 YFP-G3BP1 CEs were treated with 18U micrococcal nuclease (+) or left untreated (-) prior to the translation reaction, and subsequently treated with 250 nM dsRNA before embedding in CEODs. (**a**) Representative images (scale bar, 20 µm). (**b**) Median YFP-G3BP1 condensate area (top panel). The number of analysed CEODs (n) from 2 biological replicates is indicated on the top. Bottom panel: percentage of CEODs with condensates (2 biological replicates). Statistical analysis (Kruskal-Wallis, Dunn's multiple comparisons test) compared to - nuclease is indicated.

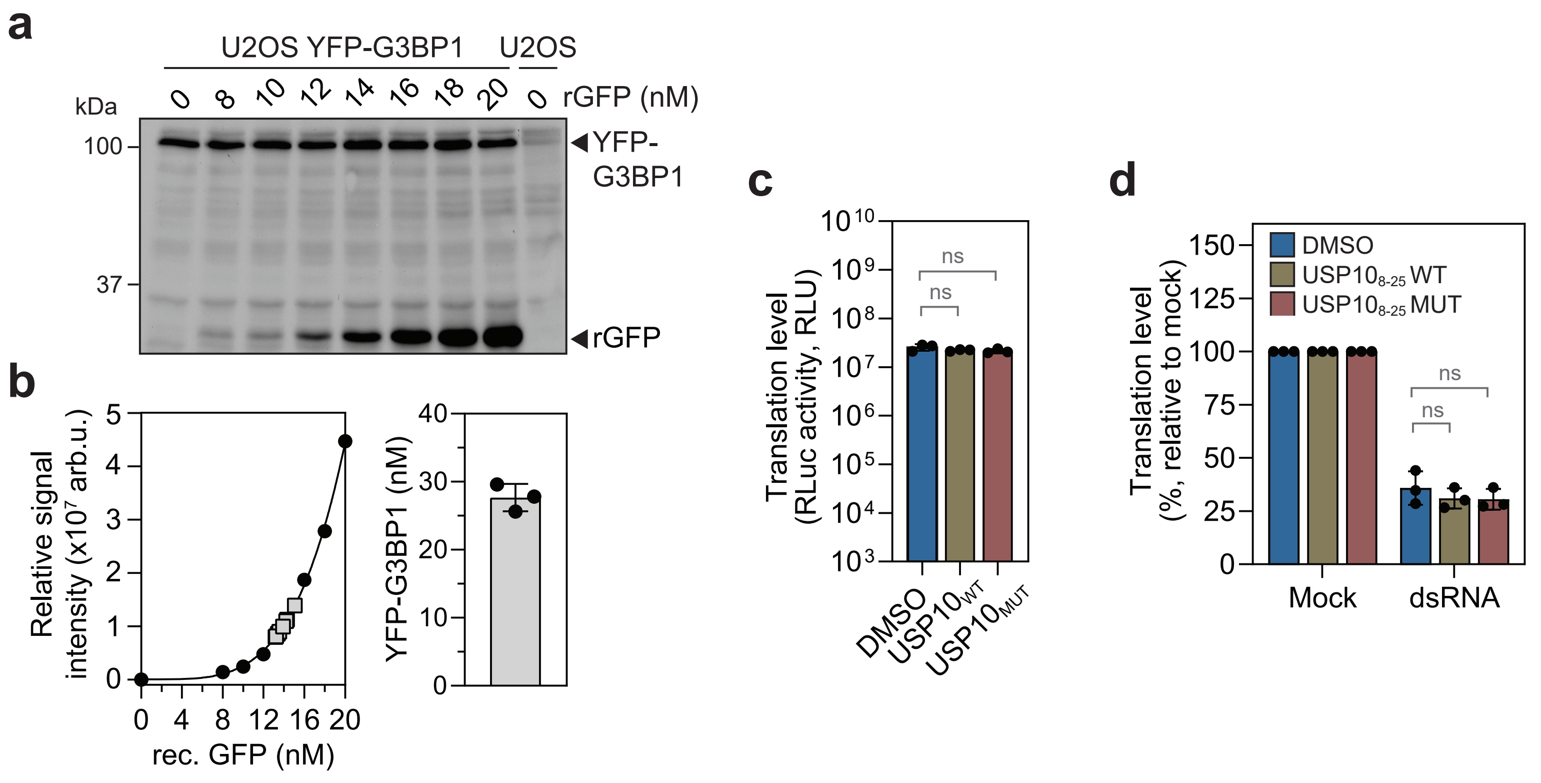

Supplementary Fig. 10. Inhibition of YFP-G3BP1 condensation by the USP10 peptide. (**a**) Quantification of YFP-G3BP1 concentration in U2OS YFP-G3BP1 CEs using quantitative Western blotting. CEs were spiked with increasing concentrations of recombinant GFP (rGFP) (n=3 independent biological replicates). Shown is a representative Western blot. (**b**) Corresponding quantifications and interpolation with the rGFP signal intensity. A mean YFP-G3BP1 concentration of 27 nM was estimated. (c and d) U2OS YFP-G3BP1 CEs were treated with vehicle control (DMSO), 10 µM USP108-25 peptide with wild-type sequence (WT) or mutated sequence (F10A-F13A) (n=3 independent biological replicates). (**c**) Translation levels of the treated CEs (mean ± SD). Statistical significance (1-way ANOVA, Tukey's multiple comparisons test) compared to DMSO is indicated. (**d**) Relative translation levels in CEs additionally treated with 250 nM dsRNA. Values (mean ± SD) are represented as percentage relative to mock CE. Statistical analysis (2-way ANOVA, Sidak's multiple comparisons test) compared to mock is indicated. ns, non-significant ($p \geq 0.05$).

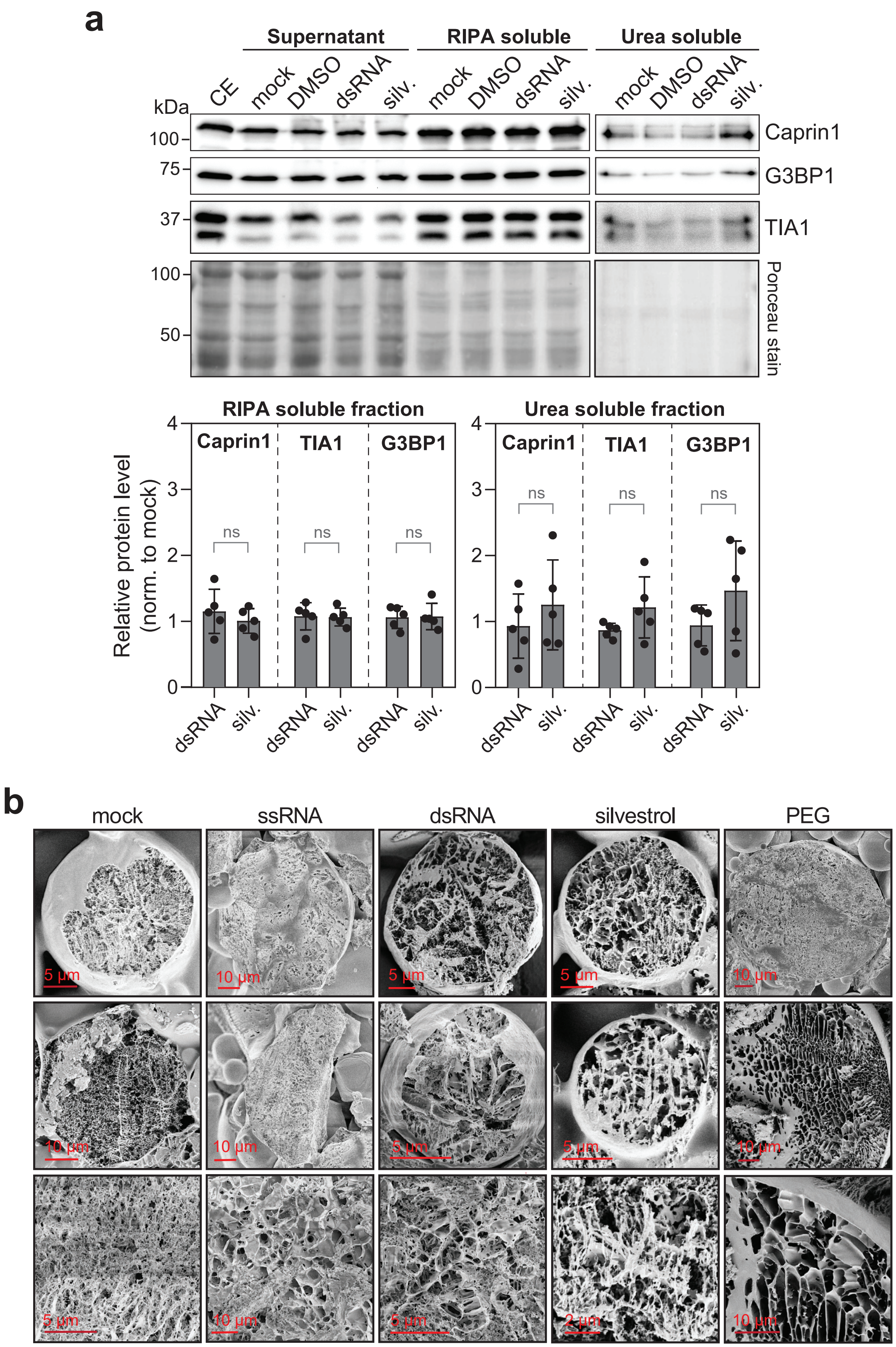

Supplementary Fig. 11. SG protein solubility and ultrastructural features of YFP-G3BP1 condensates. (**a**) U2OS YFP-G3BP1 CEODs were treated with 250 nM dsRNA, 2 µg/ml silvestrol, DMSO (vehicle control), or left untreated (mock). Induced condensates were pelleted and resuspended in RIPA buffer. The insoluble fraction was subsequently pelleted and dissolved in urea buffer. Shown is a representative Western blot analysis of the SG proteins Caprin1, G3BP1 and TIA1, in the corresponding fractions (top panel) and corresponding quantifications (bottom panel). Protein levels were normalised to total protein input as determined by Ponceau S staining. Relative expression levels were calculated relative to mock (for dsRNA) or DMSO (for silvestrol) conditions (n=5 independent biological replicates). Statistical significance (2-way ANOVA, Sidak's multiple comparisons test) compared to dsRNA is indicated. ns, non-significant ($p \geq 0.05$). (**b**) Cryo-SEM analysis of U2OS CEODs treated with 250 nM dsRNA, 250 nM ssRNA, 2 µg/ml silvestrol or 1% PEG. Shown are representative images and magnification of CEOD regions.

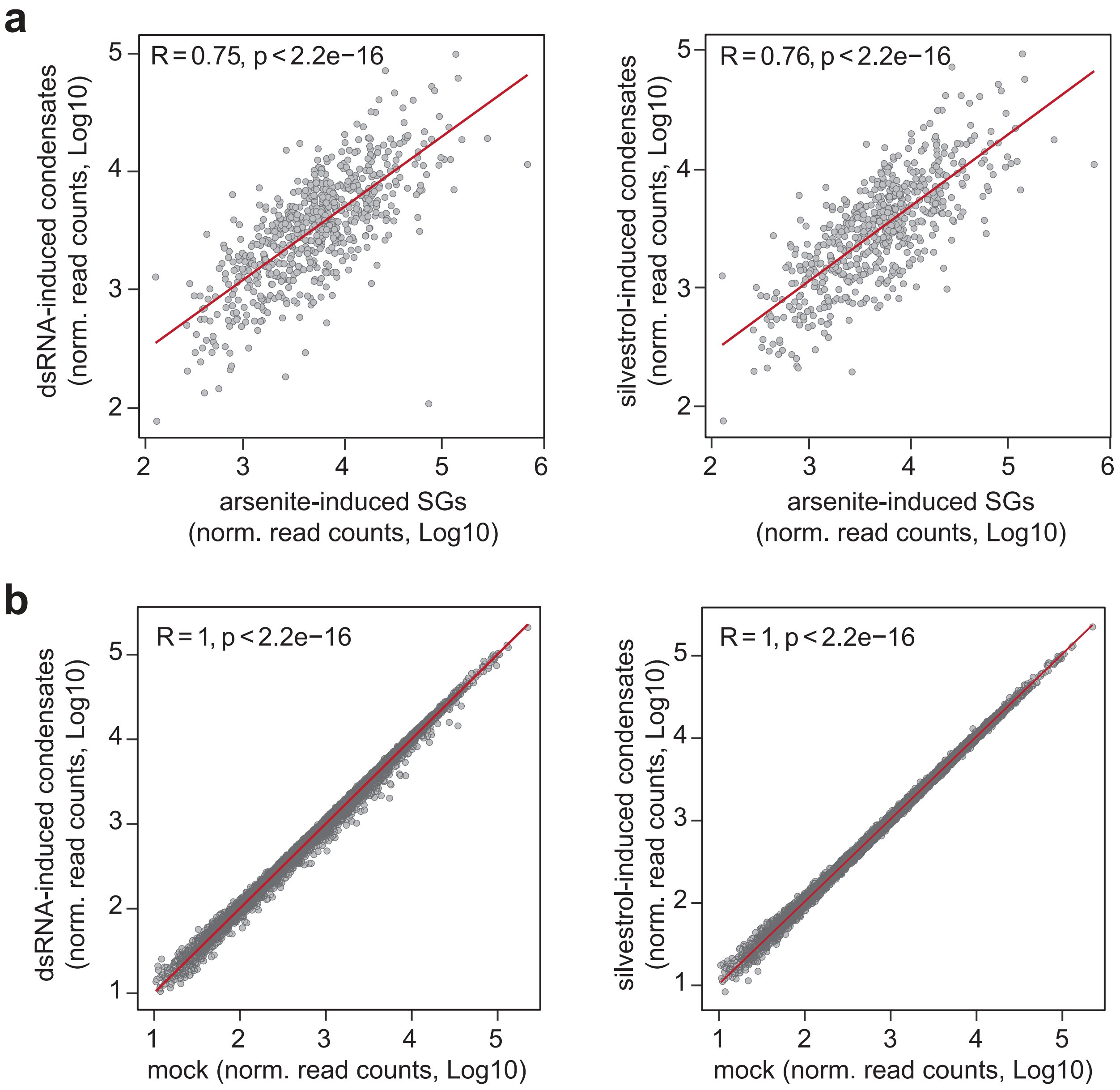

Supplementary Fig. 12. Condensate transcriptomes. YFP-G3BP1 condensates induced by dsRNA or silvestrol were pelleted and total RNA extracted for further poly(A) analysis by RNA sequencing. Scatter plots show normalised read counts (Log10) based on the DeSeq2 normalisation. Pearson's correlation coefficient (R) and p-values are indicated. (**a**) Shown are mRNAs identified in both previously published transcriptome of arsenite-induced SGs (Khong *et al.*, 2017) and transcriptomes of condensates induced by dsRNA- (671 mRNAs) or silvestrol (599 mRNAs) (fold change > 1, q-value < 0.05) (related to Fig. 8c). (**b**) Comparison of mock condensate transcriptome with the transcriptomes of dsRNA- or silvestrol-induced condensates.

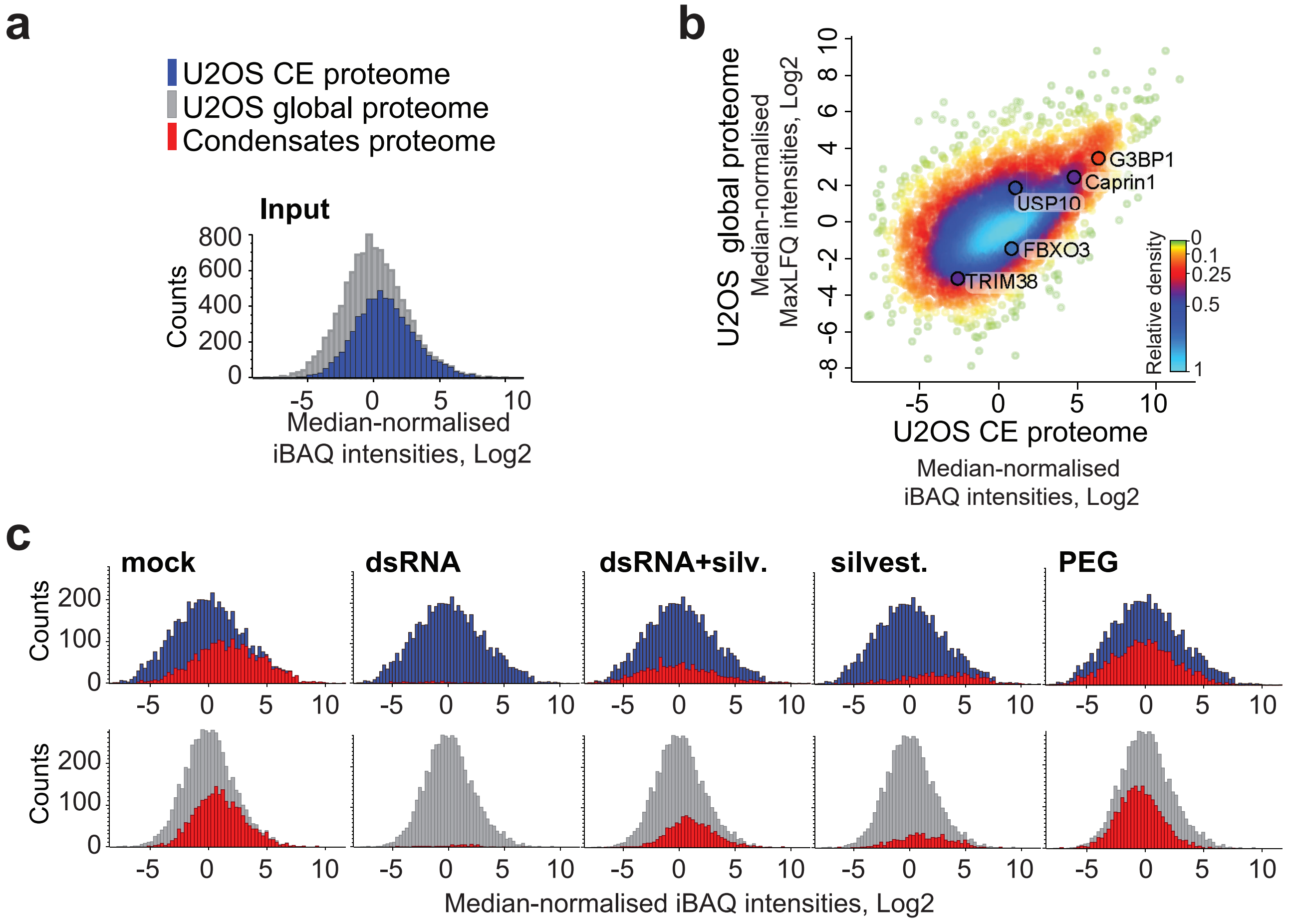

Supplementary Fig. 13. Condensate proteomes. Median-normalised Log2-transformed protein intensities from the input sample (U2OS CE proteome) and from the condensates (condensate proteome) were compared with those from a publicly available deep global proteome of U2OS cells (U2OS global proteome, PRIDE identifier: PXD045003). (**a**) Histogram of median-normalised Log2 protein intensities for all human proteins quantified in U2OS global proteome (grey). The blue histogram indicates all the proteins identified in the CE proteome (this study) within this distribution. (**b**) Density plot representation of the median-normalised CE proteome vs. U2OS global proteome. The blue-shades indicate high-density and highly correlated proteins. Highlighted are known (G3BP1, Caprin1 and USP10) and newly identified SG proteins (FBOX3 and TRIM38). (c) Histogram of median-normalised Log2 protein intensities of proteins identified in each respective condensate proteome (red bars) compared to CE proteome (top panel) or U2OS global proteome (bottom panel) distribution.

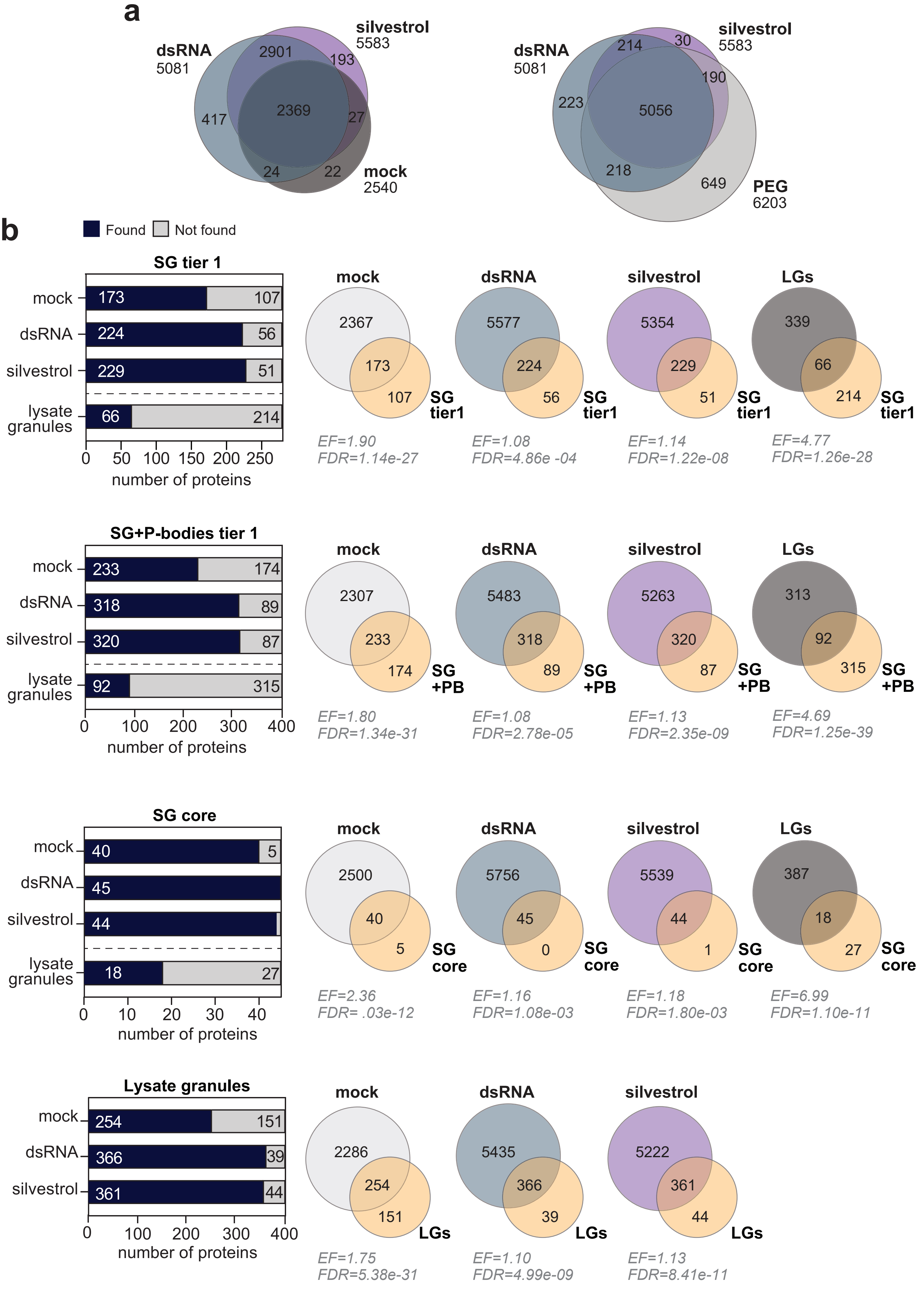

Supplementary Fig. 14. Enrichment of known SG proteins in condensates. (**a**) Venn diagram of unique and overlapping proteins identified by LC-MS/MS analysis in dsRNA-, silvestrol-, and PEG-induced condensates. Untreated samples (mock) were pelleted as control. Proteins were considered as candidate component if their respective intensities were consistently identified across all replicates of each category (at least n=4 independent technical replicates/condition). (**b**) Bar graphs and Venn diagrams showing the overlap between condensate proteomes and proteins listed in the RNA Granule Database: high-confidence and validated SG proteins “SG tier 1” (280 proteins); high-confidence “SG and P-bodies tier 1” proteins (407 proteins). Additionally, condensate proteomes were compared to a subset of “SG core” proteins (45 proteins), and to the proteome of “lysate granules” (405 proteins) (Freibaum *et al*., 2021). Statistical significance (one-sided Fisher’s exact test, enrichment factor (EF), Benjamini–Hochberg FDR Log10) are indicated at the bottom.

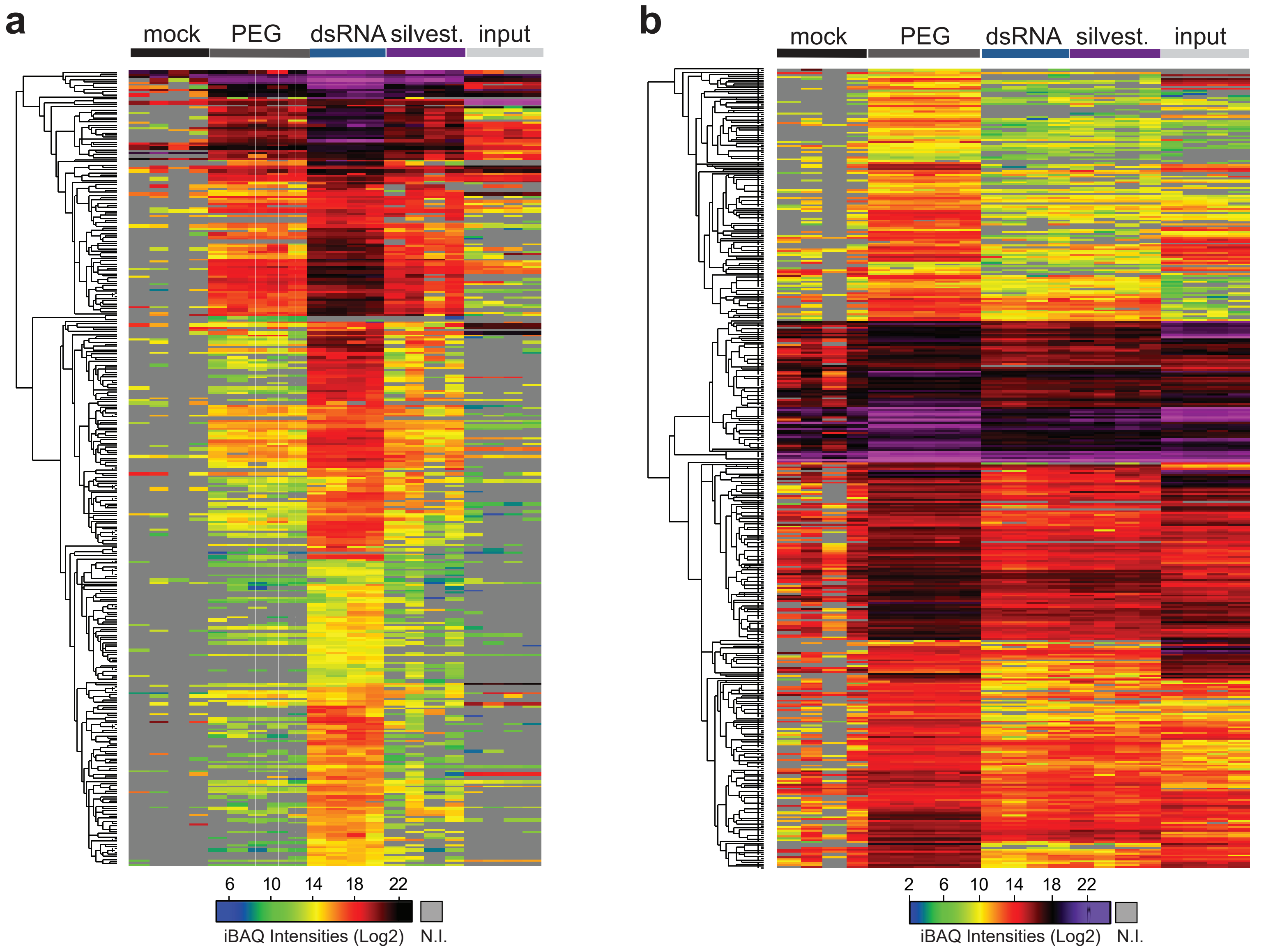

Supplementary Fig. 15. Protein abundance in condensate clusters. Supervised hierarchical clustering of the iBAQ intensity profiles (clustering based on ANOVA significance and unsupervised hierarchical cluster as in Fig. 9. Multiple-sample ANOVA, S0=0, FDR < 0.05). Shown is the global abundance of each respective protein in whole CE (before condensate enrichment). N.I., not identified. (**a**) Analysis of 315 proteins significantly enriched in dsRNA-induced condensates. (**b**) Analysis of the 2,702 proteins significantly enriched in PEG-induced condensates.

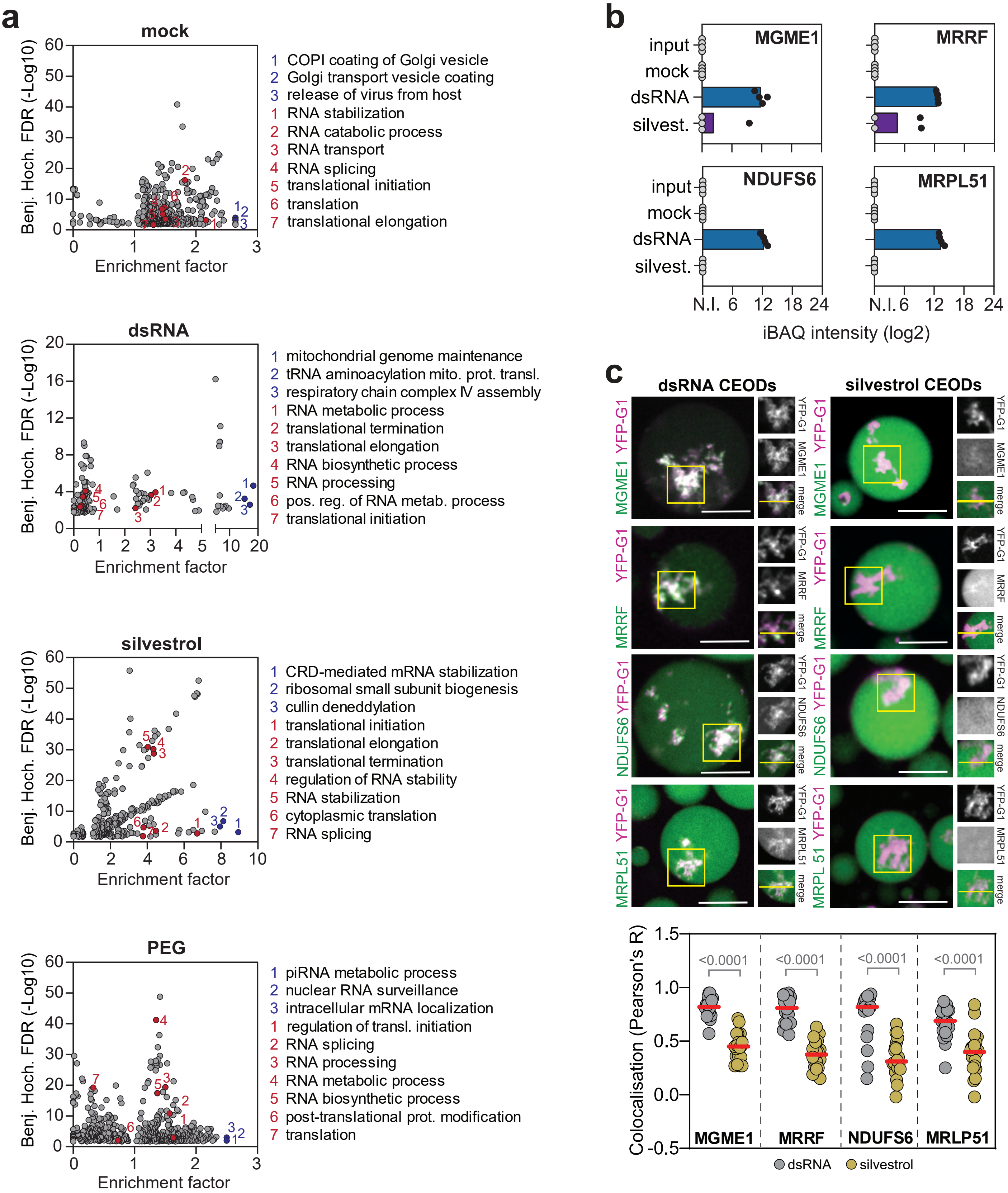

Supplementary Fig. 16. Analysis of gene ontology biological process. (**a**) Enriched gene ontology biological process (GOBP) annotations for individual clusters (clustering based on ANOVA significance and unsupervised hierarchical cluster as in Fig. 9. Multiple-sample ANOVA, S0=0, FDR < 0.05). Coloured dots represent the 3 most enriched GOBP terms (blue) and 7 GOBP terms commonly identified in the RNA Granule Database (SG tier 1) (red) are indicated (one-sided Fisher's exact test, enrichment score > 2, Benjamini–Hochberg FDR Log10 < 0.02). (b and c) Characterisation of selected candidates exclusive to the dsRNA-specific cluster. (**b**) Protein intensities (iBAQ, Log2) in condensate-enriched fractions across biological replicates and treatment groups (n=4-5). Input indicates relative abundance in CE before pelleting. Not identified proteins (N.I.) are displayed as light-grey coloured dots. (**c**) Upper panel: localisation of candidates in dsRNA- and silvestrol-treated YFP-G3BP1 CEODs. Primary antibodies were coupled to Alexa-Fluor 647-labelled secondary antibodies (magenta) prior mixing with the CEs and encapsulation in CEODs. Shown are representative images of CEOD and cropped sections of condensates (yellow squares). Scale bar, 20 µm. Bottom panel: scatter plot shows the colocalisation of candidates with YFP-G3BP1, assessed using Pearson's correlation coefficient (R), for 20 condensates induced by dsRNA (grey) and silvestrol (brown). Statistical significance (2-way ANOVA, Sidak's multiple comparisons test) compared to dsRNA is indicated. ns, non-significant (p ≥ 0.05).

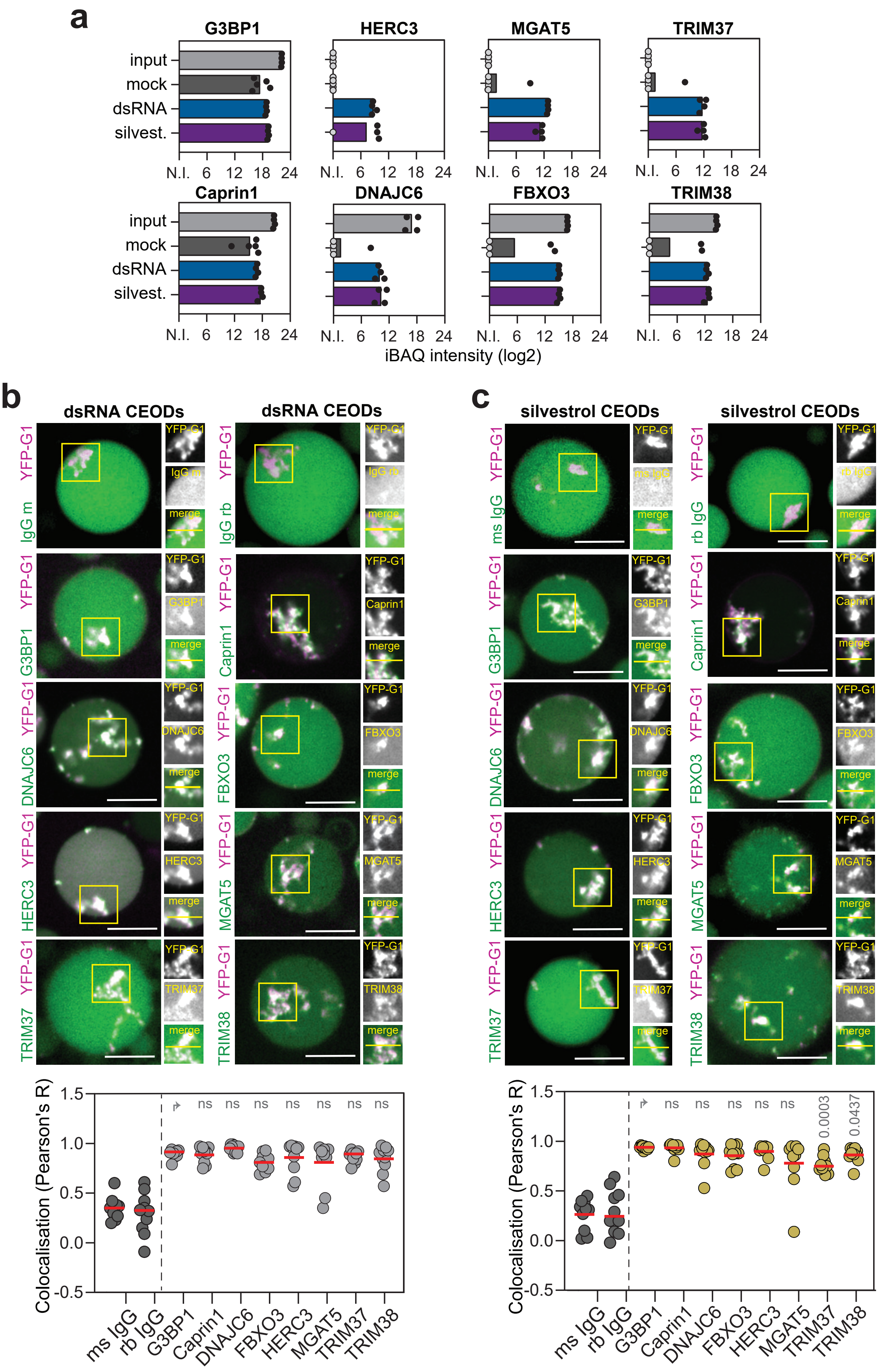

Supplementary Fig. 17. Validation of SG client proteins in CEODs. Characterisation of selected candidates from the dsRNA and silvestrol cluster. (**a**) Protein intensities (iBAQ, Log2) in condensate-enriched fractions across biological replicates and treatment groups (n=4-5). Input indicates relative abundance in whole CE before enrichment (pelleting). Not identified proteins (N.I.) are displayed as light-grey coloured dots. (**b** and **c**) Candidates identified as potential SG components were validated by immunofluorescence assay in YFP-G3BP1 CEODs. Specific antibodies targeting the SG markers G3BP1 and Caprin1 were used as positive controls. IgG mouse and IgG rabbit served as negative controls. Primary antibodies were coupled to Alexa-Fluor647-labelled secondary antibodies (magenta) prior mixing with the CEs and encapsulation in CEODs (n=10 condensates). Condensation was induced by 250 nM dsRNA (b) or 2 µg/µl silvestrol (c). Shown are representative images of CEODs stained with antibodies recognising IgG, G3BP1, Caprin1 or SG candidates and cropped sections (yellow squares) of the condensates. Scale bar, 20 µm. Scatter plots at the bottom show the colocalisation of newly identified SG proteins with YFP-G3BP1, assessed using Pearson's correlation coefficient (R), for 10 dsRNA- and 10 silvestrol-induced condensates in different CEODs. Statistical significance (1-way ANOVA, Dunnett's multiple comparisons test) compared to G3BP1 is indicated. ns, non-significant ($p \geq 0.05$).

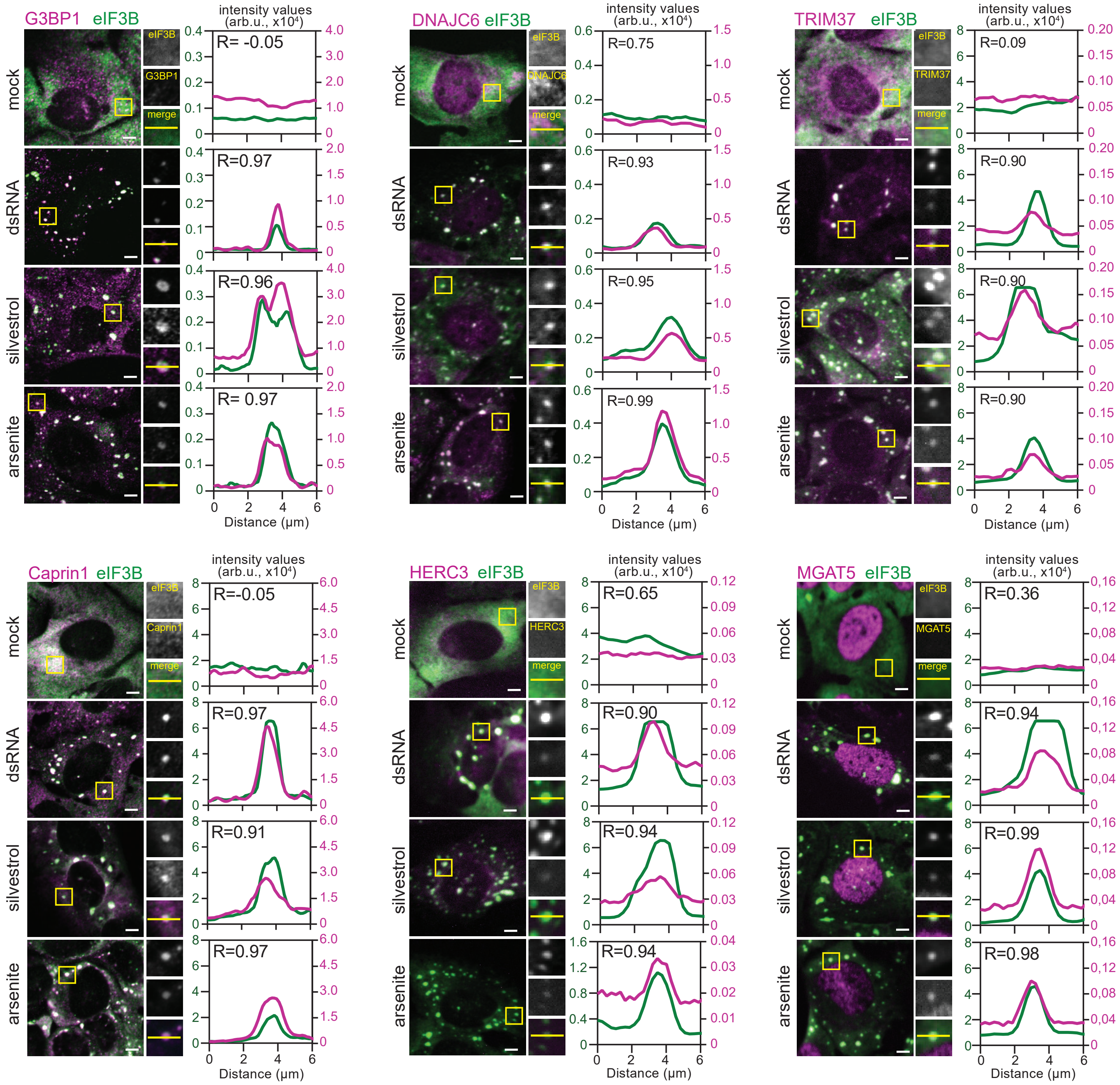

Supplementary Fig. 18. Newly identified SG client proteins localize to SGs induced by several stressors. The localisation of candidates identified by LC-MS/MS as novel components of YFP-G3BP1 condensates was analysed in U2OS cells transfected with dsRNA, treated with silvestrol, or with arsenite. Untreated cells (mock) served as control. Shown are representative images and cropped sections of SGs (yellow squares). Scale bar, 5 µm. eIF3B was used as a bona fide SG marker. G3BP1 and Caprin1 colocalisation with eIF3B is shown as comparison. Intensity profiles for eIF3B and SG client fluorescence signals are shown on the side. Colocalisation was assessed using Pearson's correlation coefficient (R), which quantifies the linear relationship between pixel intensities in the two fluorescence channels along the line indicated in the cropped section.

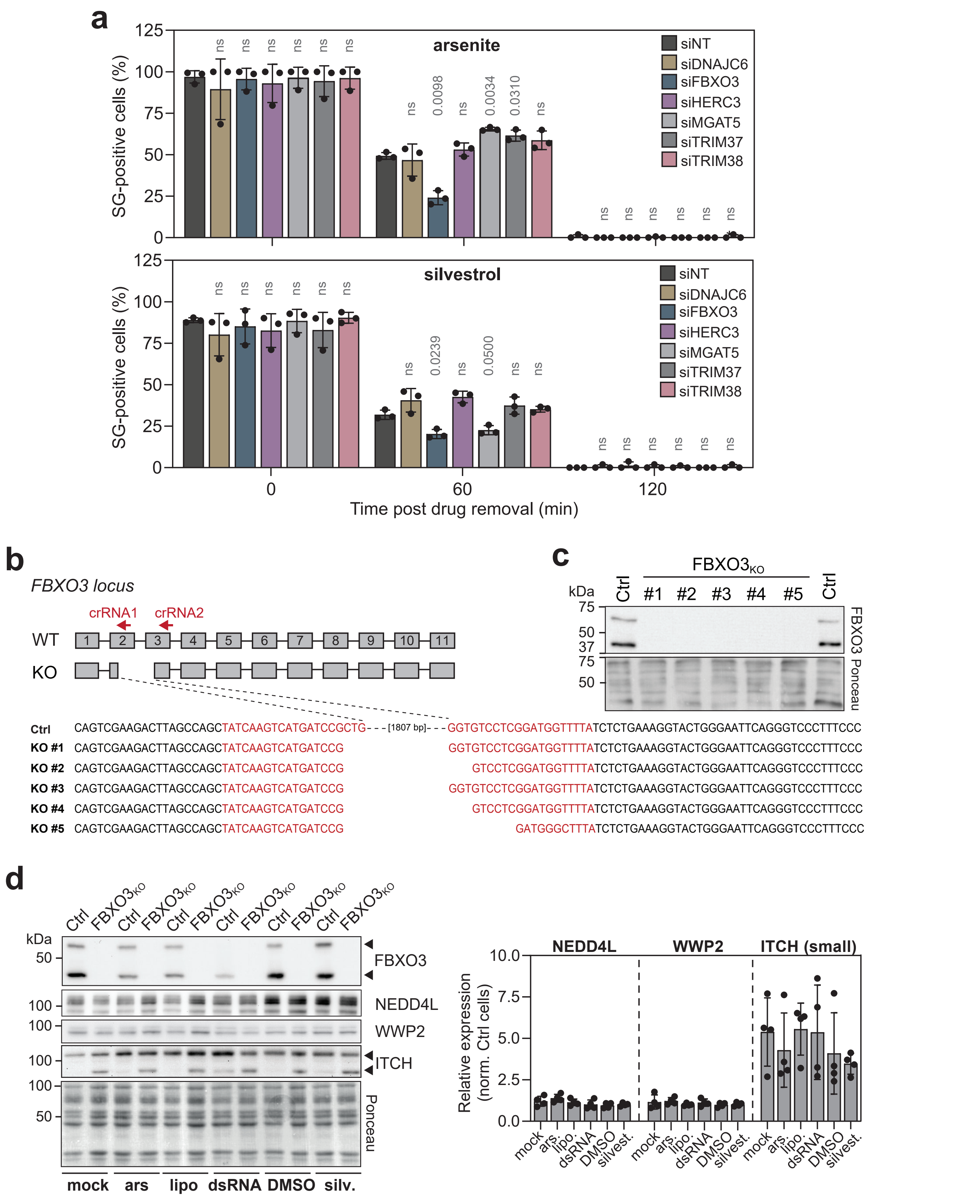

Supplementary Fig. 19. Characterisation of newly identified SG client proteins. (**a**) The expression of the novel SG clients was silenced in U2OS using siRNA pools. Cells transfected with a non-targeting control siRNA (siNT) were used as control. After 48 h, cells were treated with 0.25 mM arsenite for 45 min (upper panel) or 2 µg/ml silvestrol for 1 h (lower panel) (T0). After SG induction (T0), drugs were removed, and cells washed with PBS before addition of fresh medium for 120 min. Cells were fixed and analysed by fluorescence microscopy at the indicated time post drug removal. Shown are percentage of SG-positive cells (mean ± SD, n=3 biological replicates). Statistical significance is shown compared to siNT (2-way ANOVA, Dunnett's multiple comparisons test). (**b** and **c**) Generation of U2OS FBXO3$_{KO}$ cell clones. (b) Schematics of the human *FBXO3* locus (WT). A sequence spanning exon 2 to 3 was deleted using two CRISPR guide RNAs (crRNA1 and crRNA2). Five homozygous clones were selected based on the deletion of the genomic DNA sequence between the crRNAs and confirmed by sequencing. Cells treated with a non-targeting crRNA were used as control (Ctrl). Nucleotide deletions are indicated by dashes. (c) Analysis of FBXO3 expression in Ctrl and KO cell clones. Shown is a representative Western blot analysis. (**d**) Analysis of the expression of a selection of proteins from the NEDD4 protein family in U2OS Ctrl and FBXO3$_{KO}$. Pools of five FBXO3$_{KO}$ and Ctrl cell clones were treated with arsenite (ars.), transfected with dsRNA, or treated with silvestrol (silv.) and the corresponding controls. Shown is a representative Western blot analysis (n=4 independent biological replicates) (left panel) and corresponding quantifications of protein expression levels (right panel). Relative protein levels were normalized to Ctrl cells. Note that only the ITCH small isoform is upregulated in U2OS FBXO3$_{KO}$ cells. ns, non-significant ($p \geq 0.05$).

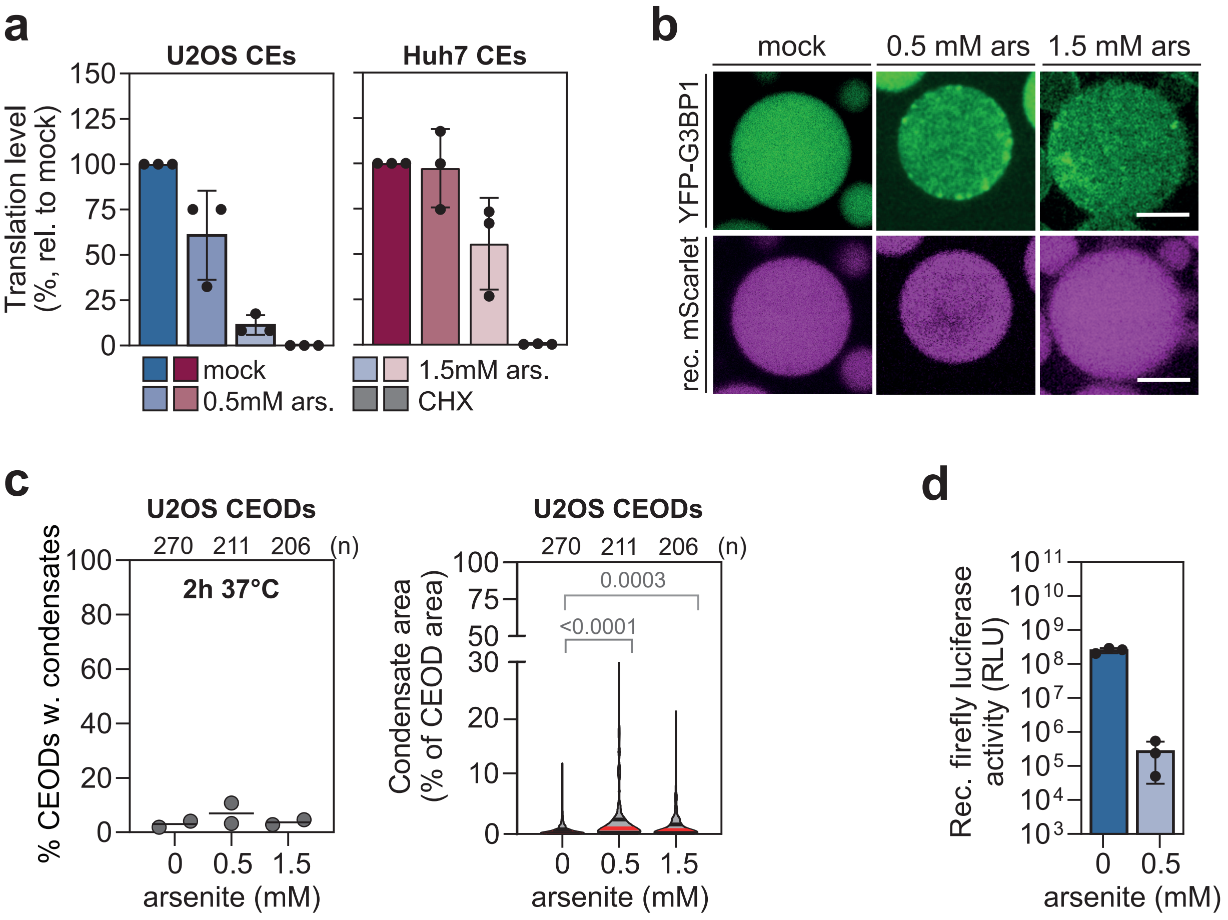

Supplementary Fig. 20. Sodium arsenite is not a suitable inducer of LLPS in CEODs. (**a**) Relative translation levels of RLuc transcript in U2OS CEs (left panel) and Huh7 CEs (right panel) treated with increasing concentrations of sodium arsenite (ars.). Values (mean ± SD, n=3 biological repeats) are represented as percentage relative to untreated. (**b**) Representative images of U2OS YFP-G3BP1 CEODs treated with increasing concentrations of arsenite. Scale bar, 20 µm. (**c**) Percentage of CEODs with condensates (left panel, 2 biological replicates) and median YFP-G3BP1 condensate area (right panel). The number of analysed CEODs (n) from 2 biological replicates and statistical significance (Kruskal-Wallis test, Dunn's multiple comparison test) compared to 0 mM arsenite are indicated. (**d**) Recombinant firefly luciferase activity in U2OS CEs treated with 0.5 mM arsenite or left untreated (mean ± SD, n=3 biological repeats).

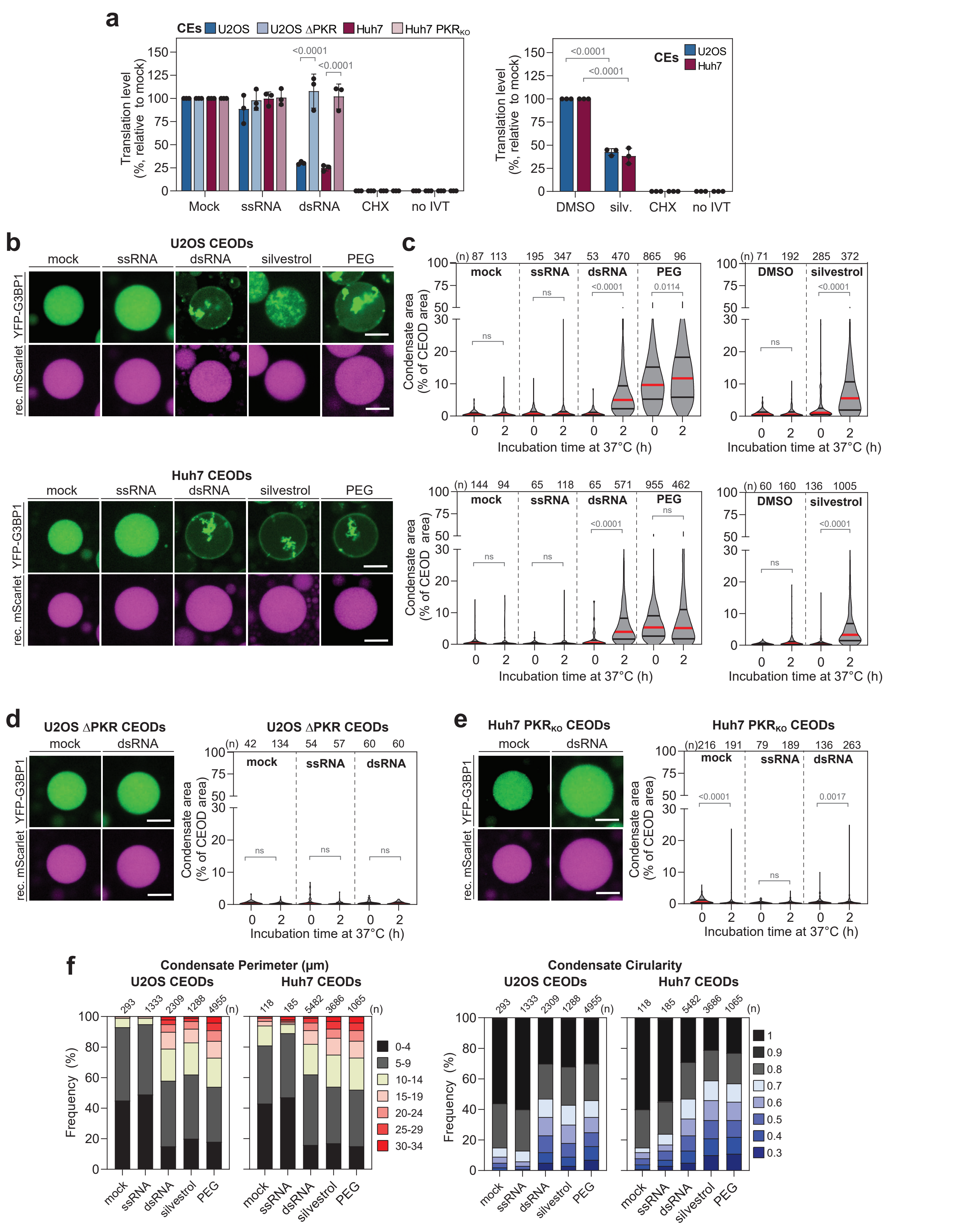

Supplementary Fig. 21. Validation of new cell lines expressing the corrected YFP-G3BP1. The sequence of YFP-G3BP1 containing its original stop codon was inserted in a lentiviral vector. U2OS, U2OS ΔPKR, Huh7 and Huh7 PKR_{KO} YFP-G3BP1 cells were generated by lentiviral transduction. Translation levels and condensate formation were analysed for each cell line (n=3 independent biological replicates). (**a**) Translation level of RLuc transcript in CEs after 2 h incubation at 37 °C with 250 nM dsRNA (left panel) or 2 µg/ml silvestrol (right panel). Values (mean ± SD) are represented as percentage relative to untreated CEs (mock) (2-way ANOVA, Tukey's multiple comparisons test). CEs treated with ssRNA and CHX were used as control. (**b** and **c**) YFP-G3BP1 CEODs were treated with 250 nM dsRNA, 250 nM ssRNA, 2 µg/ml silvestrol or 1% PEG. (b) Representative images of U2OS YFP-G3BP1 CEODs (upper panel) and Huh7 YFP-G3BP1 CEODs (lower panel). Scale bar, 20 µm. (c) Median YFP-G3BP1 condensate area in U2OS CEODs (upper panel) and Huh7 CEODs (lower panel). The number of analysed CEODs (n) from 2 biological replicates is indicated on the top (Kruskal-Wallis test, Dunn's multiple comparison test). (**d** and **e**) U2OS ΔPKR CEODs (d) and Huh7 PKR_{KO} CEODs (e) were treated with 250 nM dsRNA. Shown are representative images of U2OS ΔPKR and Huh7 PKR_{KO} CEODs (left panels). Scale bar, 20 µm. Right panels: median YFP-G3BP1 condensate area in CEODs. The number of analysed CEODs (n) from 2 biological replicates is indicated on the top. Stastistical significance (Kruskal-Wallis test, Dunn's multiple comparisons test) compared to 0 h is indicated. ns, non-significant ($p \geq 0.05$). (**f**) YFP-G3BP1 condensates' features. Bar graphs depict the frequency of condensates with specified perimeter (left panels) and circularity (right panels) in U2OS and Huh7 YFP-G3BP1 CEODs. The number of analysed CEODs from 2 biological replicates is indicated on the top.

		U2OS cells	CEODs
**Confinement size**	Diameter (median)	19.6 ± 3.1 µm	25.3 ± 13.1 µm
	Diameter range	12.6 - 28.6 µm	15-100 µm
	Volume (fl)	3.9 ± 0,6 fl	8.5 ± 4.4 fl
**Protein concentration**	µg/µl	128 ± 20 µg/µl	5 µg/µl
	per cell or CEOD (median)	0.5 ± 0.2 ng	0.04 ± 0.02 ng
**RNA concentration**	ng/µl	5,848 ± 929 ng/µl	46.7 ± 3.7 ng/µl
	per cell or CEOD (median)	22.9 ± 0.6 pg	0.39 ± 0.21 pg

Supplementary Table 1. Comparison of parameters between cells and CEODs. The table presents median confinement sizes, protein concentrations (in µg/µl), RNA concentrations (in ng/µl), and total protein and RNA amounts per individual cell or CEOD (in pg or ng, as indicated). Protein and RNA amount per CEOD was used to calculate the median diameter. Protein and RNA amounts per cell were estimated by measuring bulk protein and RNA concentrations from a fixed number of cells in 3 biological replicates.

			Condensates	SGs
**Perimeter**	induced by dsRNA	mean ± SD	6.2 ± 5.0 µm	7.0 ± 5.9 µm
		range	0.5 - 74.1 µm	2.5 - 48.8 µm
	induced by silvestrol	mean ± SD	7.5 ± 5.8 µm	4.4 ± 3.5 µm
		range	0.5 - 58.7 µm	1.0 - 49.5 µm
**Circularity**	induced by dsRNA		0.7 ± 0.2	0.8 ± 0.1
	induced by silvestrol		0.8 ± 0.2	0.8 ± 0.2

Supplementary Table 2. Comparison between condensates and SGs. The table summarizes the mean perimeter and size range of condensates induced by dsRNA and silvestrol, and their circularity in U2OS condensates and SGs.

Supplementary Fig. 22. Uncropped scans presented in this study. Indicated in red are the molecular weight markers (MW). The red dotted boxes represent the cropped sections show in the respective figures.

Scans related to Fig. 2b

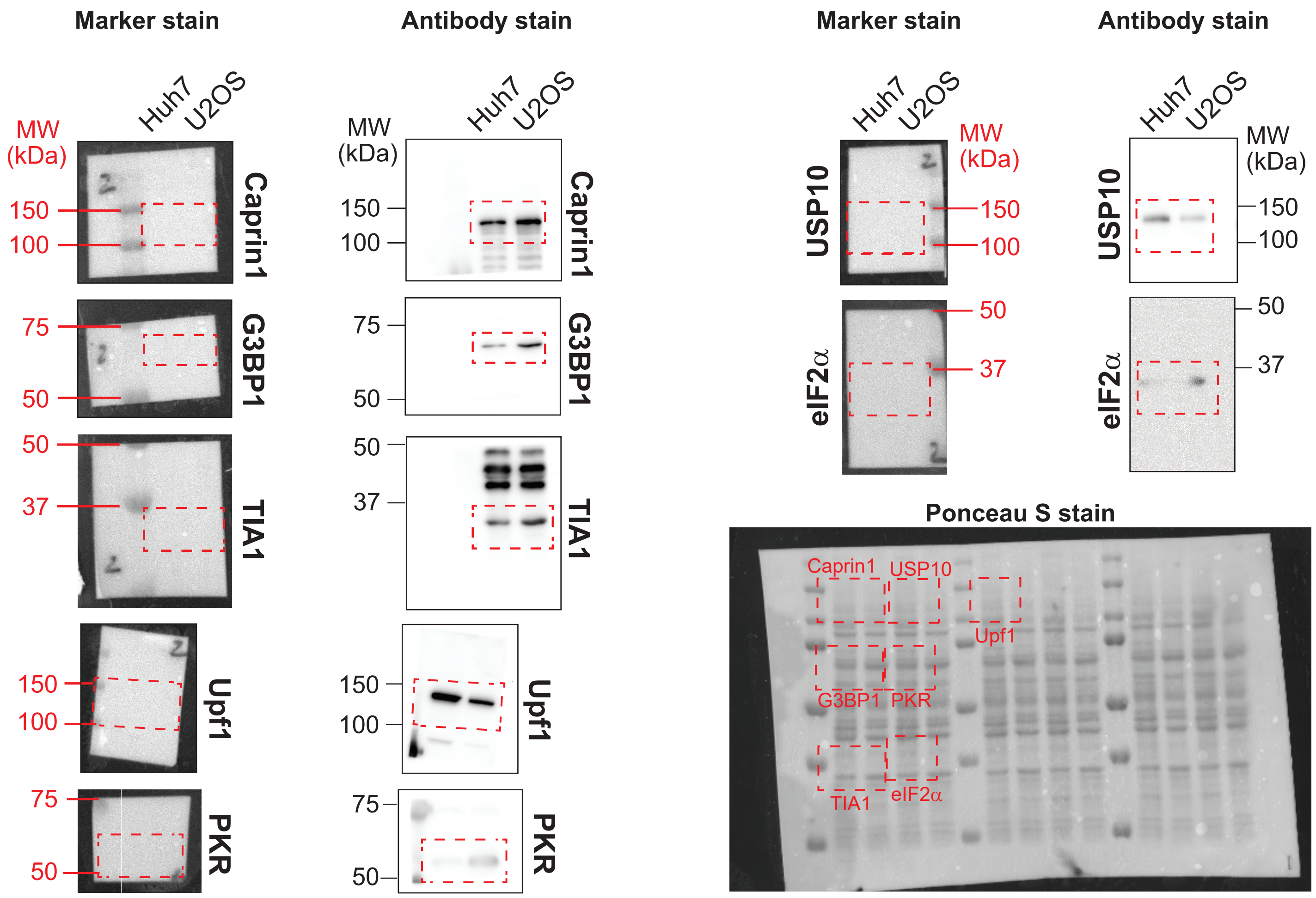

Scans related to Supplementary Fig. 2b

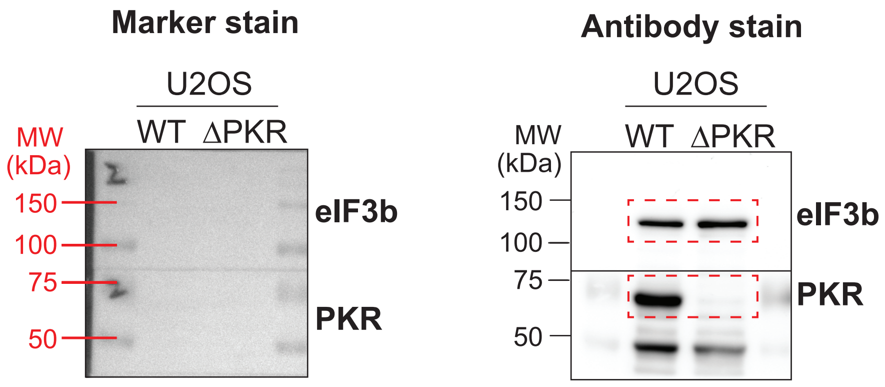

Scans related to Supplementary Fig. 2d

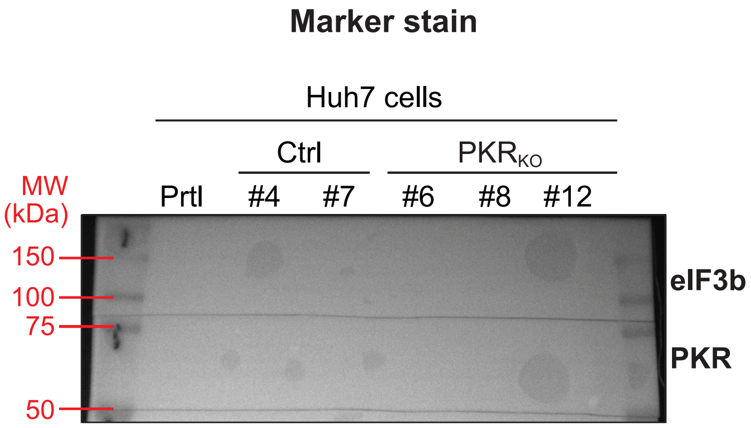

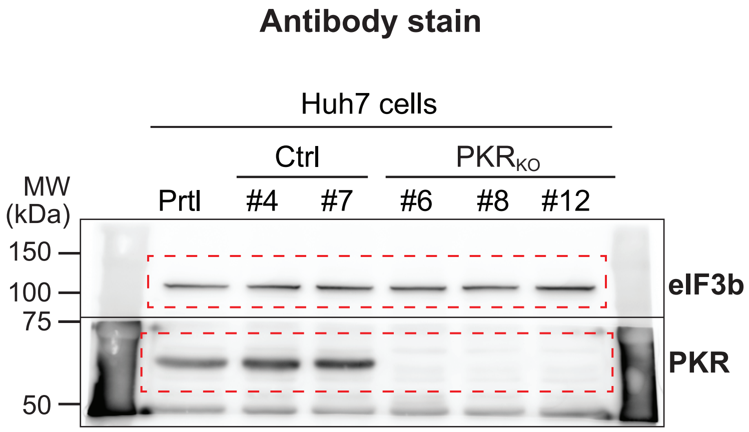

Scans related to Supplementary Fig. 8a

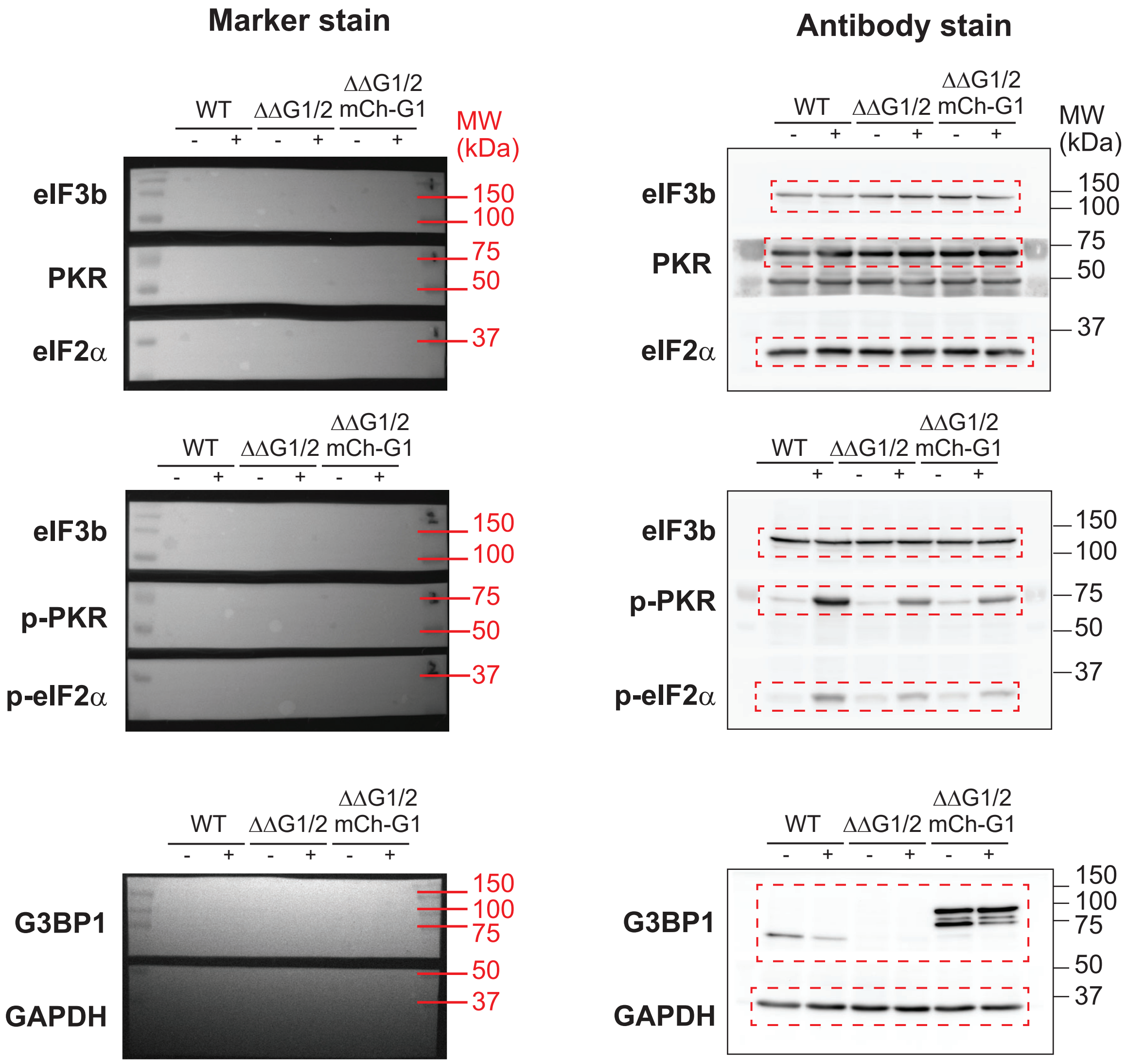

Scans related to Supplementary Fig. 10a

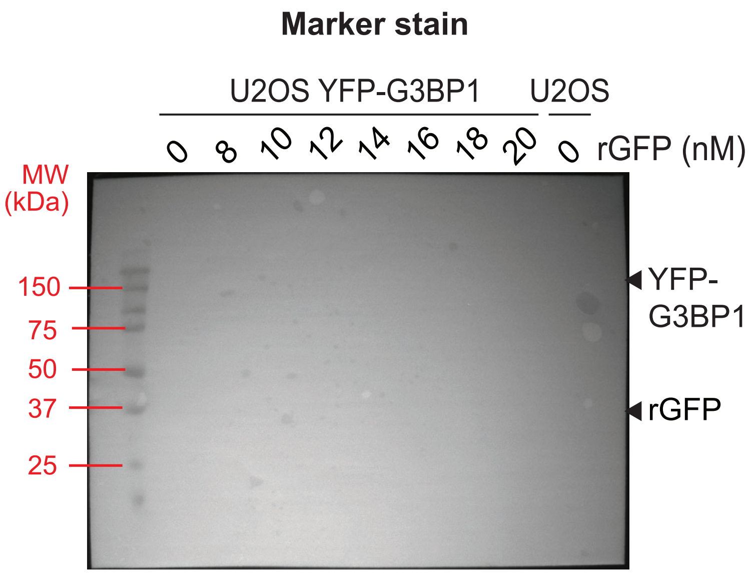

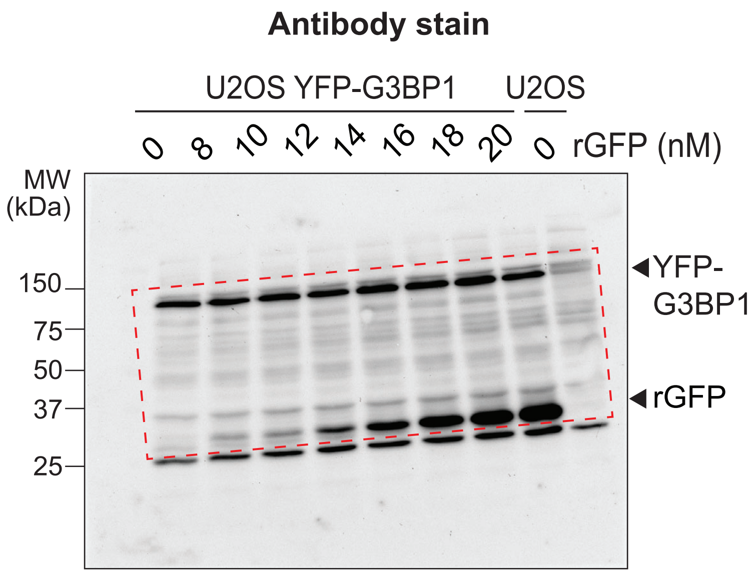

Scans related to Supplementary Fig. 11a

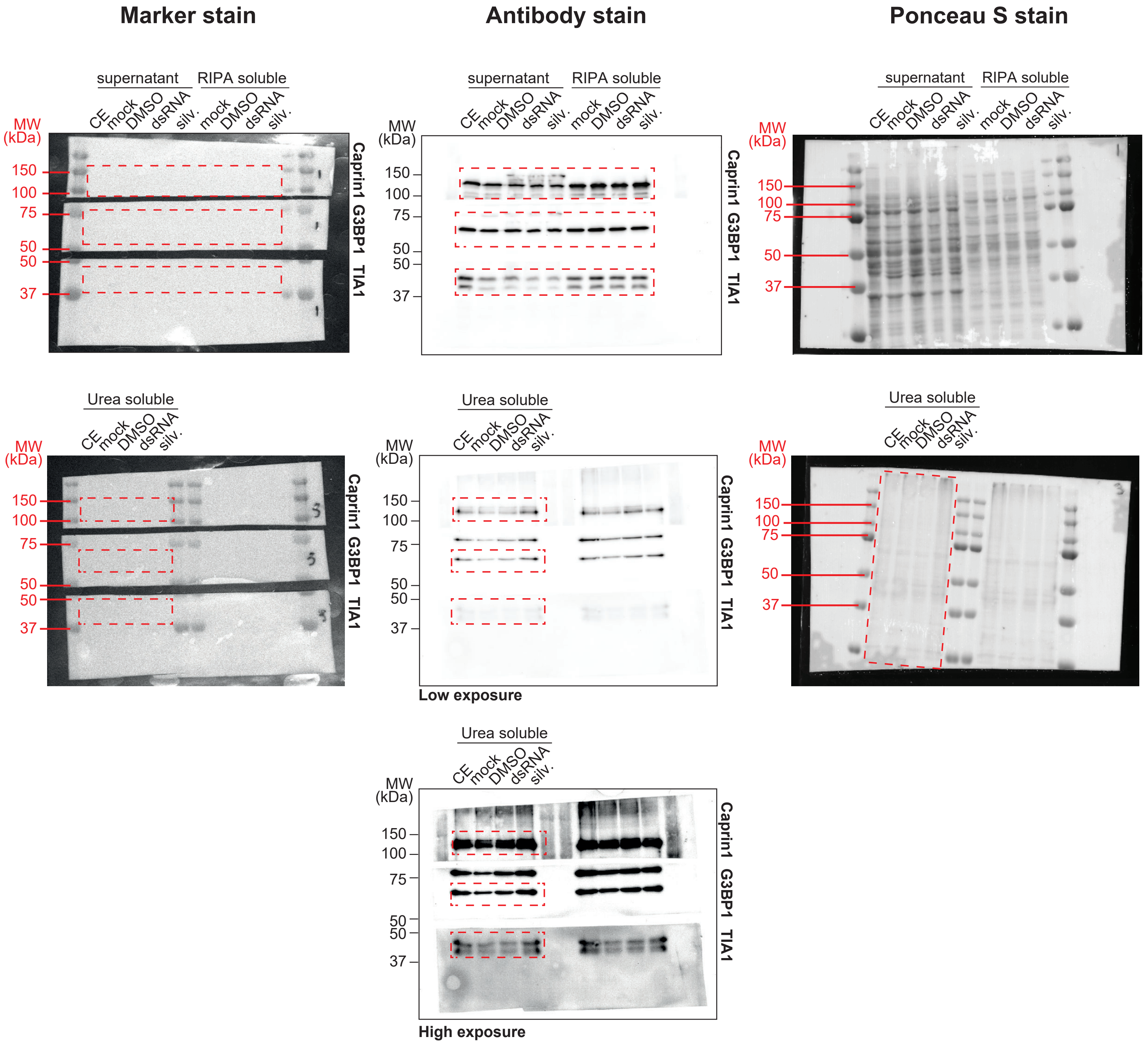

Scans related to Supplementary Fig. 11b

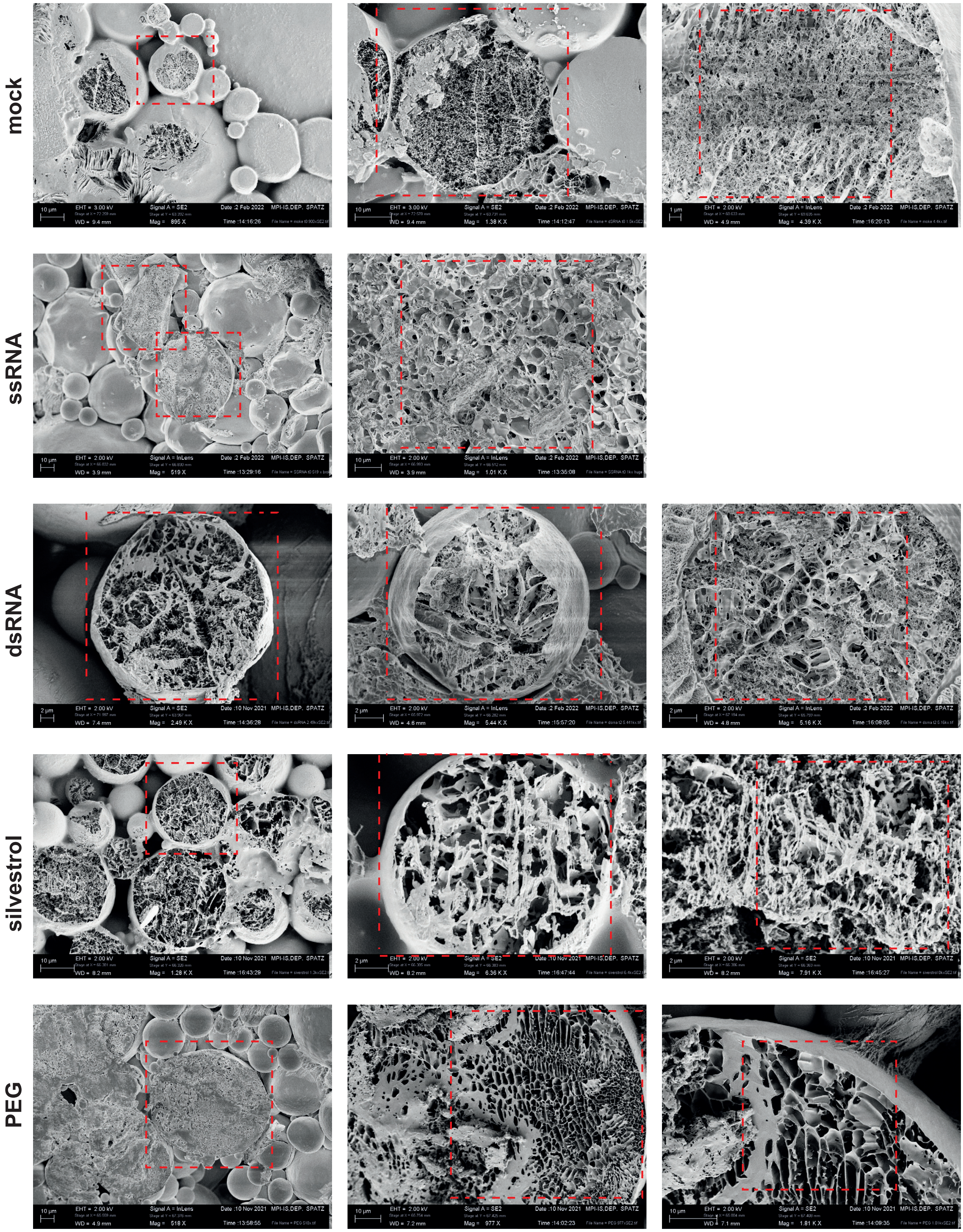

Scans related to Supplementary Fig. 19c

Marker stain

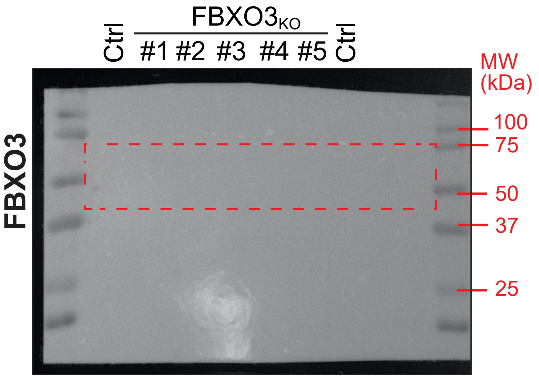

Antibody stain

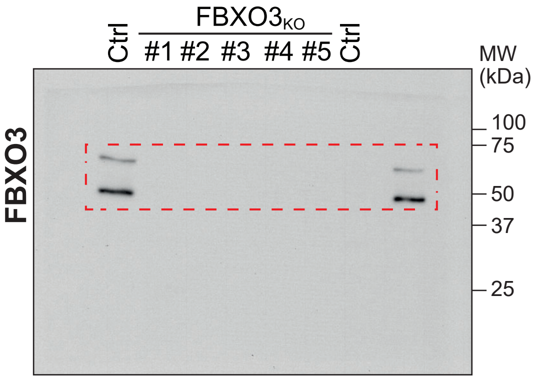

Ponceau S stain

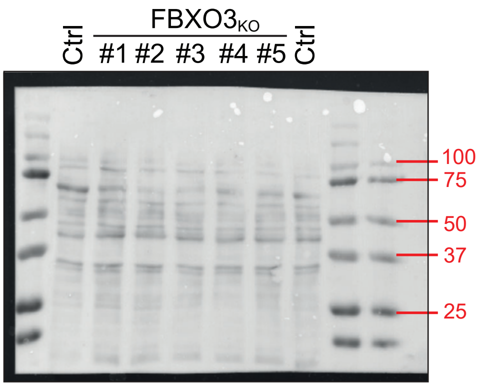

Scans related to Supplementary Fig. 19d

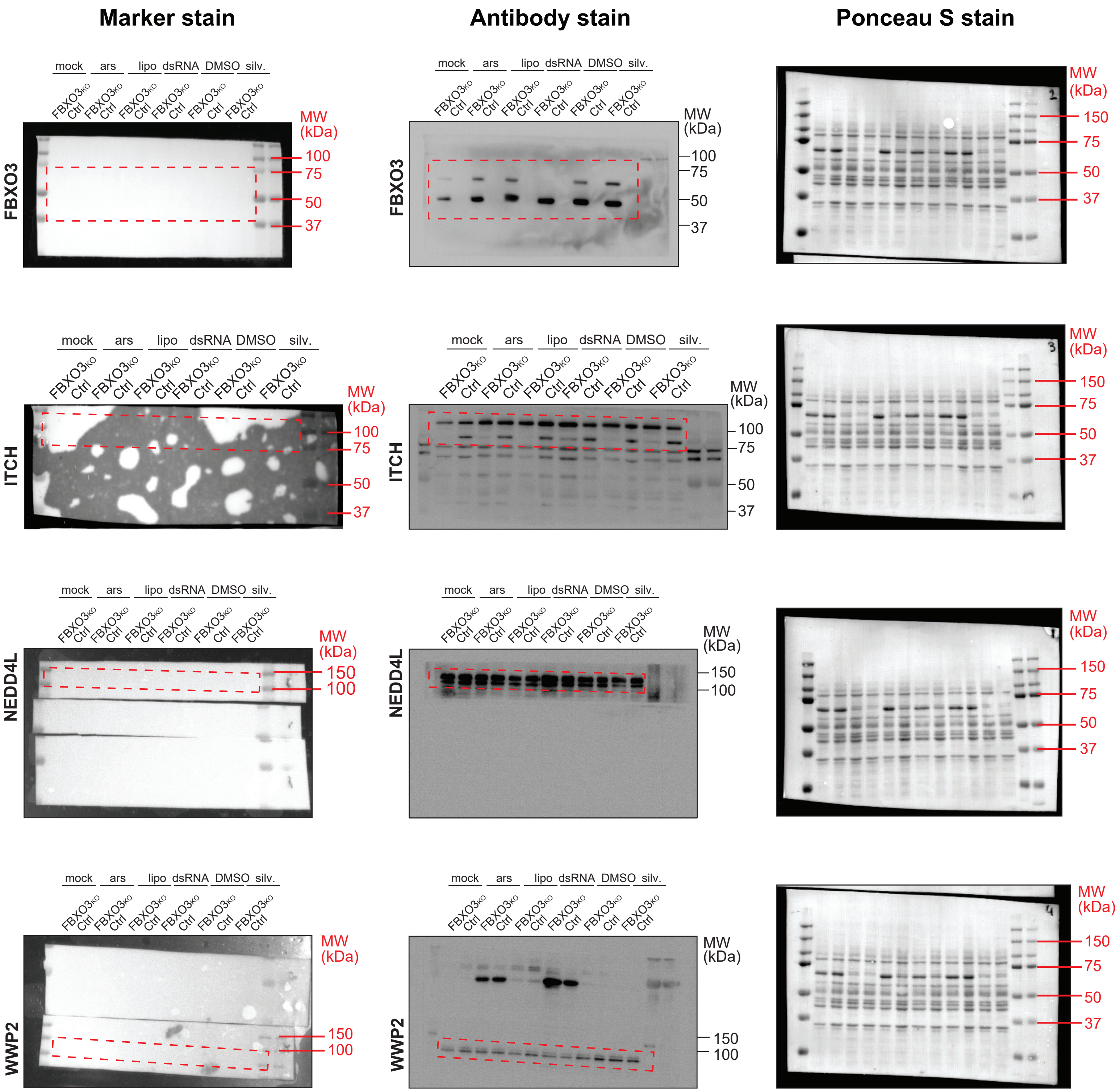

Supplement: Supplementary file 1 — Supplementary Information [file 41467_2026_73936_MOESM1_ESM.pdf]
