# Supplementary material for: Stress granule phase separation in stress-responsive cytosolic extract-in-oil droplets
Source: Nat Commun. 2026 Jun 5;17:5011. doi: 10.1038/s41467-026-73936-x (PMC13241516; doi:10.1038/s41467-026-73936-x)
Supplement: Supplementary file 2 — Description of Additional Supplementary Files [file 41467_2026_73936_MOESM2_ESM.pdf]

Title: Supplementary Data 1
Description: Condensates-RNAseq-raw-read-counts.

Title: Supplementary Data 2
Description: RNAseq-differential-expression-analysis.

Title: Supplementary Data 3
Description: Condensates-quantitative-LC-MS-proteomics.

Title: Supplementary Data 4
Description: GO-term-analysis.

Title: Supplementary Data 5
Description: Selected-hit-lists.

Title: Supplementary Movie 1
Description: Dynamics of YFP-G3BP1 condensate formation in CEODs treated with dsRNA.

Title: Supplementary Movie 2
Description: FRAP analysis of G3BP1 mobility in CEODs.
