# Supplementary material for: Stress granule phase separation in stress-responsive cytosolic extract-in-oil droplets
Source: Nat Commun. 2026 Jun 5;17:5011. doi: 10.1038/s41467-026-73936-x (PMC13241516; doi:10.1038/s41467-026-73936-x)
Supplement: Supplementary file 10 — Reporting Summary [file 41467_2026_73936_MOESM10_ESM.pdf]

nature portfolio

Corresponding author(s): Alessia, Ruggieri

Last updated by author(s): Mar 2, 2026

Reporting Summary

Nature Portfolio wishes to improve the reproducibility of the work that we publish. This form provides structure for consistency and transparency in reporting. For further information on Nature Portfolio policies, see our Editorial Policies and the Editorial Policy Checklist.

Statistics

For all statistical analyses, confirm that the following items are present in the figure legend, table legend, main text, or Methods section.

n/a	Confirmed	
☐	☒	The exact sample size (*n*) for each experimental group/condition, given as a discrete number and unit of measurement
☐	☒	A statement on whether measurements were taken from distinct samples or whether the same sample was measured repeatedly
☐	☒	The statistical test(s) used AND whether they are one- or two-sided<br>*Only common tests should be described solely by name; describe more complex techniques in the Methods section.*
☒	☐	A description of all covariates tested
☐	☒	A description of any assumptions or corrections, such as tests of normality and adjustment for multiple comparisons
☐	☒	A full description of the statistical parameters including central tendency (e.g. means) or other basic estimates (e.g. regression coefficient) AND variation (e.g. standard deviation) or associated estimates of uncertainty (e.g. confidence intervals)
☐	☒	For null hypothesis testing, the test statistic (e.g. *F*, *t*, *r*) with confidence intervals, effect sizes, degrees of freedom and *P* value noted<br>*Give P values as exact values whenever suitable.*
☒	☐	For Bayesian analysis, information on the choice of priors and Markov chain Monte Carlo settings
☒	☐	For hierarchical and complex designs, identification of the appropriate level for tests and full reporting of outcomes
☐	☒	Estimates of effect sizes (e.g. Cohen's *d*, Pearson's *r*), indicating how they were calculated

Our web collection on statistics for biologists contains articles on many of the points above.

Software and code

Policy information about availability of computer code

Data collection	Luciferase activity: ICE v.2.0.2.0 (Berthold Technologies)<br><br>Western blot: ChemoStar Touch v.0.5.84 (INTAS Science Imaging Instruments)<br><br>Microscopy:<br>- CEODs and condensates: NIS-Elements Advanced Research software v.5.21 (Nikon)<br>- Fluorescence recovery after photobleaching: Volocity v6.3 (Perkin Elmer)<br>- Fluorescence images: NIS-Elements Advanced Research software v.5.30.06 (Nikon)<br><br>Mass spectrometry: nanoElute plug-in v.1.1.0.27 (Bruker)
Data analysis	Western blot LabImage1D software (INTAS Science Imaging Instruments)<br><br>Microscopy:<br>- Image analysis pipeline (segmentation of CEODs and condensates): NIS-Elements Advanced Research software v.5.21 (Nikon).<br>- Fluorescence intensity, colocalization: FIJI software package (Schindelin et al. 2012; PMID: 22743772)<br><br>Graphing and statistical analysis: Prism v.10.4.0 (GraphPad)

Proteomics: MaxQuant software v.1.6.17.0 using the built-in label-free quantitation algorithm and Andromeda search engine (Tyanova et al., 2016; PMID: 27809316), and further processed with the Perseus software v.1.6.15.0 (Tyanova et al., 2016; PMID: 27348712)

Transcriptomics: DESeq2 package (Love et al., 2014; PMID: 25516281)

For manuscripts utilizing custom algorithms or software that are central to the research but not yet described in published literature, software must be made available to editors and reviewers. We strongly encourage code deposition in a community repository (e.g. GitHub). See the Nature Portfolio guidelines for submitting code & software for further information.

Data

Policy information about availability of data

All manuscripts must include a data availability statement. This statement should provide the following information, where applicable:

- Accession codes, unique identifiers, or web links for publicly available datasets
- A description of any restrictions on data availability
- For clinical datasets or third party data, please ensure that the statement adheres to our policy

The main data supporting the results of this study are available within the paper and the Supplementary Information.
The datasets generated and analysed during the current study are available in the Source Data file provided in this paper.
The analysis pipeline (segmentation of CEODs and condensates) is available on request for import into the Nikon NIS-Elements software.
The proteomics data have been deposited at the ProteomeXchange Consortium (http://proteomecentral.proteomexchange.org) via the PRIDE partner repository with the following dataset identifier: PXD057906. Source data are provided with this paper.
The RNA sequencing data have been deposited at the NCBI GEO and are accessible with the following identifier: GSE308184.

Research involving human participants, their data, or biological material

Policy information about studies with human participants or human data. See also policy information about sex, gender (identity/presentation), and sexual orientation and race, ethnicity and racism.

Reporting on sex and gender	This study does not involve human participants.
Reporting on race, ethnicity, or other socially relevant groupings	This study does not involve human participants.
Population characteristics	This study does not involve human participants.
Recruitment	This study does not involve human participants.
Ethics oversight	This study does not involve human participants.

Note that full information on the approval of the study protocol must also be provided in the manuscript.

Field-specific reporting

Please select the one below that is the best fit for your research. If you are not sure, read the appropriate sections before making your selection.

[x] Life sciences [ ] Behavioural & social sciences [ ] Ecological, evolutionary & environmental sciences

For a reference copy of the document with all sections, see nature.com/documents/nr-reporting-summary-flat.pdf

Life sciences study design

All studies must disclose on these points even when the disclosure is negative.

Sample size	No sample size calculation was performed.
Data exclusions	Data were not excluded.
Replication	At least two independent biological replicates were performed for each experiment.
Randomization	No randomization was used as experiments only involved cell lines.
Blinding	No blinding was done. Analyses were conducted in an automated manner using software reducing the occurrence of bias.

Reporting for specific materials, systems and methods

We require information from authors about some types of materials, experimental systems and methods used in many studies. Here, indicate whether each material, system or method listed is relevant to your study. If you are not sure if a list item applies to your research, read the appropriate section before selecting a response.

Materials & experimental systems

n/a	Involved in the study	
	☒	Antibodies
	☒	Eukaryotic cell lines
☒		Palaeontology and archaeology
☒		Animals and other organisms
☒		Clinical data
☒		Dual use research of concern
☒		Plants

Methods

n/a	Involved in the study	
☒		ChIP-seq
☒		Flow cytometry
☒		MRI-based neuroimaging

Antibodies

Antibodies used

p-eIF2α (Cell Signaling, 9721S)
eIF2α (Cell Signaling, 9722S)
PKR (Proteintech, 18244-1-AP)
p-PKR (Abcam, ab32036)
eIF3b (Bethyl Laboratories, A301-761A)
GFP (Roche, 11814460001)
G3BP1 (Bethyl Laboratories, A302-034A)
NEDD4L (Proteintech, 13690-1-AP)
WWP2 (Proteintech, 12197-1-AP)
ITCH (Proteintech, 20920-1-AP)
eIF3η (Santa Cruz, sc-137214)
eIF3B (Bethyl Laboratories, A301-761A)
G3BP1 (BD Biosciences, 611126)
Caprin1 (Proteintech, 15112-1-AP)
DNAJC6 (Abcam, ab262876)
FBXO3 (Santa Cruz, sc-514625)
HERC3 (Santa Cruz, sc-100720)
MGAT5 (Invitrogen, MA5-24325)
TRIM37 (Santa Cruz, sc-515044)
TRIM38 (Invitrogen, OTI15A3)
MGME1 (Proteintech, 23178-1-AP)
MRPL51 (Abcam, ab243821)
MRRF (Proteintech, 66942-1-Ig)
NDUFS6 (Abcam, ab195808)
donkey anti-mouse Alexa Fluor 488 (Invitrogen, A-21202)
donkey anti-rabbit Alexa Fluor 568 (Invitrogen, A-21206)
goat anti-rabbit HRP (Sigma Aldrich, A6154)
goat anti-mouse HRP (Sigma Aldrich, A4416)

Validation

All antibodies were purchased commercially and used according to manufacturer's recommendation and validated applications. Antibodies against PKR, FBXO3 and G3BP1 were validated using KO cell lines in this study.

Eukaryotic cell lines

Policy information about cell lines and Sex and Gender in Research

Cell line source(s)

- U2OS (human osteosarcoma cell line, female), CVCL_0042 (Aulas et al., 2017; PMID: 28096475)
- U2OS ΔPKR cells (Aulas et al., 2017; PMID: 28096475)
- U2OS ΔΔG3BP1/2 (Kedersha et al., 2016; PMID: 27022092)
- U2OS ΔΔG3BP1/2 mCherry-G3BP1 (Kedersha et al., 2016; PMID: 27022092)
- HEK293T (human embryonic kidney cell line, female), ATCC Cat. #CRL-3216, CVCL_0063
- Huh7 (hepatocyte-derived carcinoma cell line, male) CVCL_0336 (Ruggieri et al, 2012; PMID: 22817989)

Authentication

None of the cell lines was authenticated beyond visual inspection.

Mycoplasma contamination

All the cell lines were regularly tested and found negative for mycoplasma.

Commonly misidentified lines (See ICLAC register)	None of the cell lines used were listed in the ICLAC register.

Plants

Seed stocks	This study does not use plant material.
Novel plant genotypes	This study does not use plant material.
Authentication	This study does not use plant material.
